# Supplementary material for: Chaos in a bacterial stress response
Source: Curr Biol. Author manuscript; Available in PMC 2024 Oct 8. (PMC7616676; doi:10.1016/j.cub.2023.11.002)
Supplement: Supplementary Materials [file EMS199120-supplement-Supplementary_Materials.zip › 1-s2.0-S0960982223015166-mmc1.pdf]

Current Biology, Volume *33*

Supplemental Information

Chaos in a bacterial stress response

Divya Choudhary, Kevin R. Foster, and Stephan Uphoff

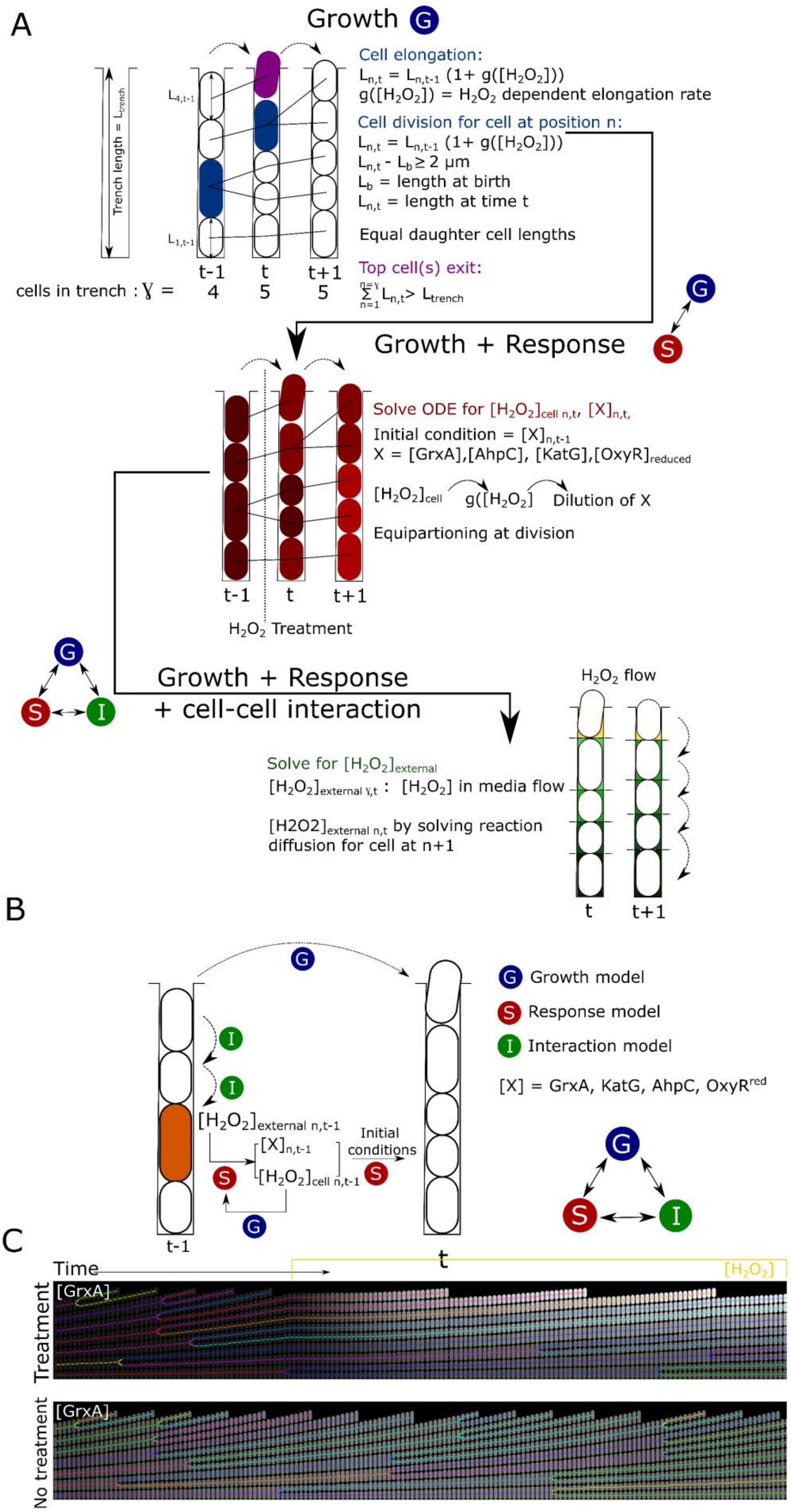
A
Growth G
Cell elongation:
L_{n,t} = L_{n,t-1} (1+ g([H2O2]))
g([H2O2]) = H2O2 dependent elongation rate
Cell division for cell at position n:
L_{n,t} = L_{n,t-1} (1+ g([H2O2]))
L_{n,t} - L_b ≥ 2 µm
L_b = length at birth
L_{n,t} = length at time t
Equal daughter cell lengths
Top cell(s) exit:
Σ L_{n,t} > L_trench
Trench length = L_trench
cells in trench : γ = 4 5 5
t-1 t t+1
Growth + Response
Solve ODE for [H2O2]_{cell n,t}, [X]_{n,t},
Initial condition = [X]_{n,t-1}
X = [GrxA],[AhpC], [KatG],[OxyR]_reduced
[H2O2]_cell g([H2O2]) Dilution of X
Equipartioning at division
H2O2 Treatment
Growth + Response
+ cell-cell interaction
H2O2 flow
Solve for [H2O2]_external
[H2O2]_{external γ,t} : [H2O2] in media flow
[H2O2]_{external n,t} by solving reaction diffusion for cell at n+1
B
Growth model
Response model
Interaction model
[X] = GrxA, KatG, AhpC, OxyR^red
[H2O2]_{external n,t-1}
[X]_{n,t-1}
[H2O2]_{cell n,t-1}
Initial conditions
C
Time
[H2O2]
[GrxA]
Treatment
No treatment

Figure S1. Illustration of model simulation with and without H_2O_2 treatment. Related to Figure 1

Illustration of the iterative procedure for simulating the inter-dependent growth, stress response, and cell-cell interaction models. (**A**) The growth model predicts the changes in the number, size, and positions of the cells in the 1-dimensional cell population for the next time point in the simulation. The output of the growth model feeds into the stress response model, which predicts for each cell the changes in the intracellular concentration of H_2O_2, concentration of reduced regulator $OxyR^{red}$, and concentrations of the stress response enzymes (GrxA, KatG, AhpCF) for the next time point. The cell elongation rate from the growth model predicts the dilution rate of enzymes. At steady-state, the average enzyme production rates and H_2O_2 influx rate are balanced with the respective average dilution rates. Temporary unbalance between production and dilution rates leads to fluctuations in concentrations in single cells. The cell-cell interaction model predicts the external H_2O_2 concentration that each cell is exposed to. A reaction-diffusion equation is solved for the uptake of H_2O_2 by the outermost cell first which is exposed to the fixed concentration of H_2O_2 in the growth media. This leads to a reduced external H_2O_2 concentration for the cell located immediately beneath the outermost cell. The procedure is repeated to predict the external H_2O_2 concentration from one cell to the next until the mother cell is reached at the bottom of the population. The outputs of the interdependent models at one time point are used as input conditions for the next time point. The simulation runs for a set number of time points. (**B**) Schematic representation for solving the growth G, response S and cell-cell interaction I models simultaneously for a representative focal cell shown in orange. I computes the $[H_2O_2]_{external}$ for the focal cell. This feeds into S solving for the concentrations of different enzymes [X] that are regulated by OxyR (e.g. GrxA, KatG, AhpC, $OxyR_{red}$) and $[H_2O_2]_{cell}$. $[H_2O_2]_{cell}$ is used as input for G to modulate the elongation rate of cell. The outputs serve as initial condition for the next time point. (**C**) Representative kymographs produced by the model for a single 1-dimensional cell population tracked over time. Greyscale shows GrxA concentration with constant 260 µM H_2O_2 treatment (top; duration of treatment marked in yellow) and without treatment (bottom).

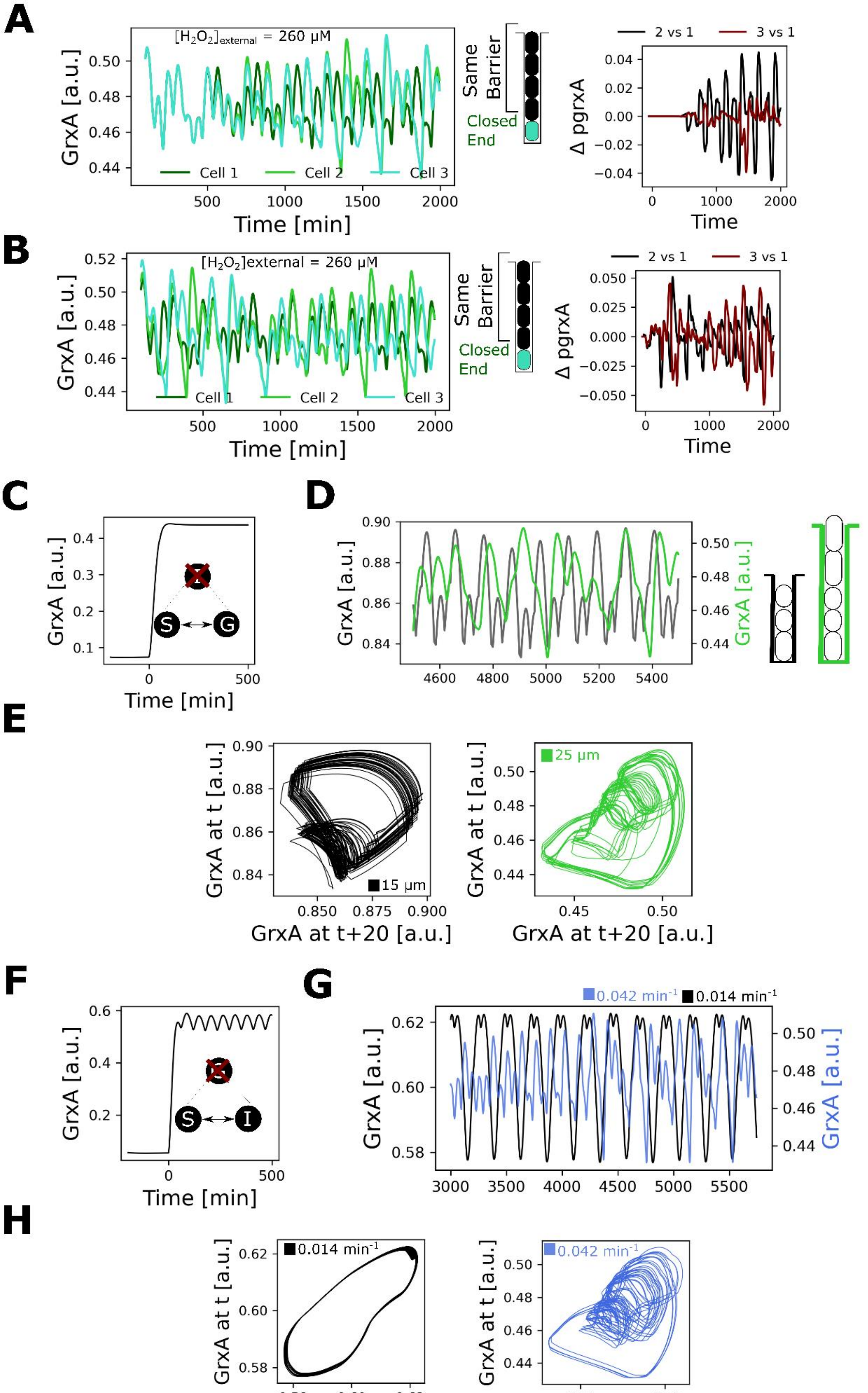
A
$[H_2O_2]_{external}$ = 260 μM
GrxA [a.u.]
Time [min]
Cell 1
Cell 2
Cell 3
Same Barrier
Closed End
2 vs 1
3 vs 1
Δ pgrxA
Time
B
$[H_2O_2]$external = 260 μM
C
GrxA [a.u.]
Time [min]
S
G
D
GrxA [a.u.]
E
GrxA at t [a.u.]
GrxA at t+20 [a.u.]
15 μm
25 μm
F
S
I
G
0.042 min⁻¹
0.014 min⁻¹
H

Figure S2. Chaotic traces of simulated stress response trajectories diverge with small differences in initial conditions and require strong coupling between stress response and cell-cell interactions or growth rate. Related to Figures 2 and 6.

(**A, B**) Left: Simulated trajectories of GrxA concentration in mother cells under constant 260 µM H_2O_2 treatment. The simulations were performed for mother cells that differ very slightly in their stage of the cell cycle at the start of the simulation. All other parameters and initial conditions were identical for the three simulations (including the number and sizes of the other cells in the population). (**A**) Mother cell length difference of $+2.5 \cdot 10^{-8}$ % (cell 2) and $+5 \cdot 10^{-8}$ % (cell 3). (**B**) Mother cell length difference of 2.5 % (cell 2) and 5% (cell 3). Right: The plots show the difference in GrxA concentration between mother cell pairs (cell 2 vs 1, cell 3 vs 1). The larger difference in initial conditions for panel B compared to panel A leads to a faster divergence of trajectories. (**C**) Response fluctuations are no longer chaotic if simulations are performed such that cell-cell interactions are absent. In this case, the concentration $[H_2O_2]_{external}$ is constant and identical for all cells in a trench. Plot shows steady GrxA expression for mother cell with 10 µM H_2O_2 treatment starting at t = 0 min. (**D**) Representative simulated GrxA traces of mother cells from the full S+G+I model (with cell-cell interactions causing $[H_2O_2]_{external}$ gradients). Periodic dynamics are seen for cells growing in a shorter growth trench with smaller population (15 µm, 2 – 4 cells, black) and chaotic dynamics for cells growing in a longer trench with larger population (25 µm, 5 – 7 cells, light green). (**E**) Phase diagrams of the GrxA traces in panel D showing closed orbits for periodic oscillations (left) but not for chaotic fluctuations (right). (**F**) Response fluctuations are no longer chaotic if simulations are performed such that growth rate is unaffected by H_2O_2 ($g([H_2O_2])$ is constant). This change uncouples the G model from the S and I models. Plot shows periodic oscillations in GrxA expression for mother cell with 400 µM H_2O_2 treatment starting at t = 0 min. (**G**) Representative simulated GrxA traces of mother cells from the full S-G-I model (with growth rate dependent on H_2O_2). Periodic dynamics are seen at low growth rate (0.014 min^{-1}, black) and chaotic dynamics at high growth rate (0.042 min^{-1}, light blue). (**H**) Phase diagrams of the GrxA traces in panel G showing closed orbits for periodic oscillations (left) but not for chaotic fluctuations (right).

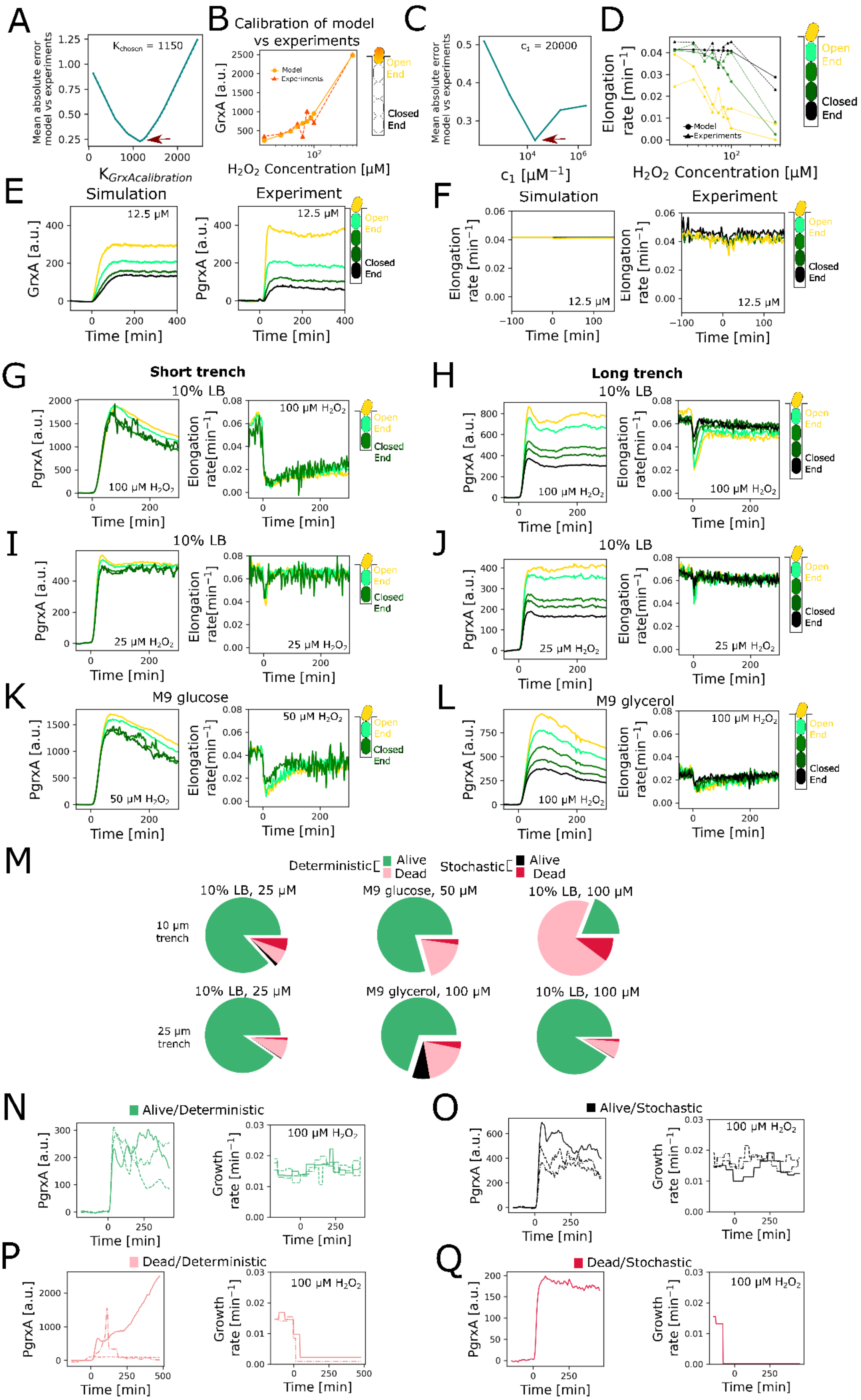
A
Mean absolute error model vs experiments
Kchosen = 1150
KGrxAcalibration
B
Calibration of model vs experiments
GrxA [a.u.]
Model
Experiments
H2O2 Concentration [μM]
Open End
Closed End
C
c1 = 20000
c1 [μM−1]
D
Elongation rate [min−1]
Model
Experiments
H2O2 Concentration [μM]
E
Simulation
Experiment
12.5 μM
GrxA [a.u.]
PgrxA [a.u.]
Time [min]
F
Simulation
Experiment
Elongation rate [min−1]
12.5 μM
Time [min]
G
Short trench
10% LB
PgrxA [a.u.]
100 μM H2O2
Elongation rate[min−1]
Time [min]
H
Long trench
10% LB
I
10% LB
25 μM H2O2
J
10% LB
K
M9 glucose
50 μM H2O2
L
M9 glycerol
100 μM H2O2
M
Deterministic
Alive
Dead
Stochastic
Alive
Dead
10% LB, 25 μM
M9 glucose, 50 μM
10% LB, 100 μM
10 μm trench
10% LB, 25 μM
M9 glycerol, 100 μM
10% LB, 100 μM
25 μm trench
N
Alive/Deterministic
100 μM H2O2
Growth rate [min−1]
O
Alive/Stochastic
P
Dead/Deterministic
Q
Dead/Stochastic

Figure S3. Stress response dynamics, elongation rate changes, and survival for different growth and H_2O_2 treatment conditions. Related to Figure 4 and 5.

Calibrating model parameters: (**A**) Mean absolute error for experimental P*grxA*-SCFP3 intensities versus GrxA concentration in model simulations for different values of calibration factor $\boldsymbol{k_{GrxA,calibration}}$. The arrow indicates the minimum point of the curve that was chosen as the parameter for all other simulations. (**B**) Experimental mean P*grxA*-SCFP3 (dashed dark orange; triangles) and calibrated GrxA values from the model (orange; circles) of cells at the open end of the trenches at steady-state for different H_2O_2 concentrations. (**C**) Mean absolute error of the cell elongation rate from experimental data versus model simulations for different values of calibration factor $\boldsymbol{c_1}$. The arrow indicates the minimum point of the curve that was used for parameter $\boldsymbol{c_1}$. (**D**) Mean cell elongation rates from model simulations (circles) and experiments (dashed; triangles) for cells at different positions in the trench over a range of H_2O_2 concentrations (yellow to black color represents cells from open to closed end of a growth trench). (**E, F**) Modelling predictions and experimental data for mean GrxA expression (left) and mean elongation rates (right) under constant 12.5 µM H_2O_2 treatment in M9 glucose from t=0 min for cells at different positions in growth trench (n = 20 simulated trenches and 3 experimental repeats). (**G-L**) Summary of oxidative stress response dynamics in experiments with different growth and H_2O_2 treatment conditions. Cells growing in shorter trenches have reduced population size, increased stress response expression, and prolonged inhibition of growth with H_2O_2 treatment compared to cells in long trenches. Mean P*grxA*-SCFP3 expression (left) and elongation rate (right) for cells growing in M9 glucose +10% LB in (**G**) 10 µm and (**H**) 25 µm trenches with 100 µM H_2O_2 treatment starting at t = 0 min (colour indicates cell position in the trench from yellow at open end to green/black at closed end of the trench, n = 3 experimental repeats). (**I, J**) Same as for panels G and H but with 25 µM H_2O_2 treatment starting at t = 0 min (n=3 experimental repeats). (**K, L**) Same as for panels G and H but with 50 µM H_2O_2 and 100 µM H_2O_2 treatment for cells growing in M9 glucose and M9 glycerol respectively (n = 3 experimental repeats). (**M**) Pie-charts indicate the fraction of dead (pink / red) and alive (green / black) mother cells classified as having deterministic (green / pink) or stochastic (black / red) stress response dynamics (based on P*grxA*-SCFP3 signal) for t > 60 min after start of H_2O_2 treatment according to the chaos decision tree algorithm by Toker *et al.* Cells were grown in different media (M9 glucose + 10% LB, M9 glucose, M9 glycerol) inside long (25 µm) or short (10 µm) growth trenches, treated with low (25 µM) or high (100 µM) H_2O_2 (on average 1249 ± 457 cells (std) with n ≥ 3 experimental repeats per pie-chart).

(**N-Q**) Representative traces of P*grxA*-SCFP3 (left) and growth rate (right) for mother cells in 25 μm growth trenches treated with 100 μM H_2O_2 at t = 0 min predicted as (**N**) alive and deterministic (green); (**O**) alive and stochastic (black); (**P**) dead and deterministic (pink); and (**Q**) dead and stochastic (red). For traces predicted as dead, the time of cell death is when growth rate drops abruptly to zero.

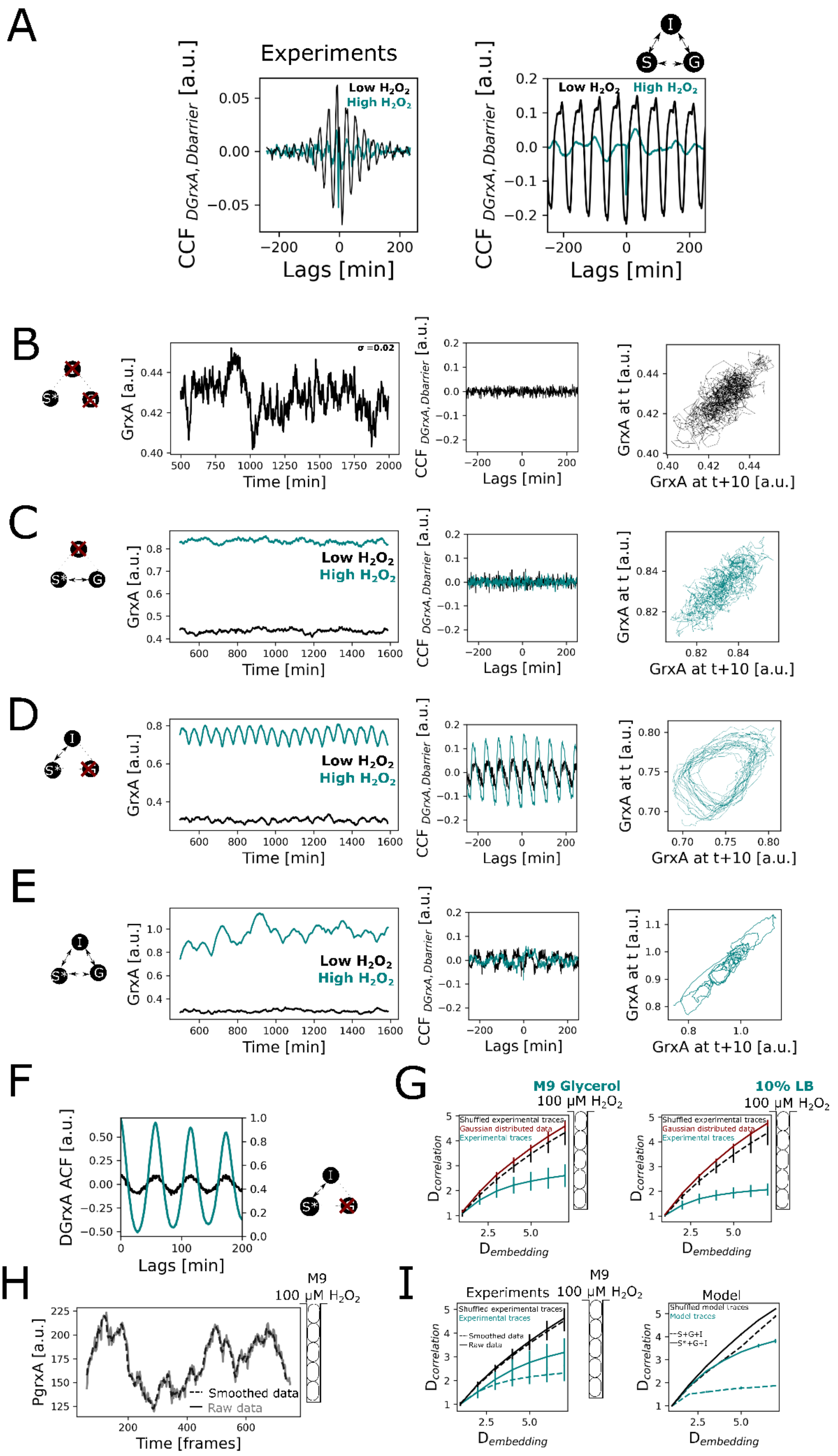

A
Experiments
Low H2O2
High H2O2
CCF DGrxA, Dbarrier [a.u.]
Lags [min]
I
S
G
B
σ =0.02
GrxA [a.u.]
Time [min]
GrxA at t [a.u.]
GrxA at t+10 [a.u.]
C
D
E
F
DGrxA ACF [a.u.]
G
M9 Glycerol
10% LB
100 μM H2O2
Shuffled experimental traces
Gaussian distributed data
Experimental traces
Dcorrelation
Dembedding
H
M9
PgrxA [a.u.]
Smoothed data
Raw data
Time [frames]
I
Experiments
Model
Shuffled model traces
Model traces
S+G+I
S*+G+I

Figure S4. Response fluctuations are not driven by noise. Related to Figure 5.

(**A**) Response fluctuations are generated by a simple cyclic cell growth pattern that generates oscillations in the number of barrier cells. Fluctuations in GrxA traces for mother cells are anti-correlated with changes in the number of barrier cells over time. Mean cross-correlation between changes in GrxA (DgrxA) and number of barrier cells (Dbarrier) for mother cells in experiment (left) and model (right) for periodic (black, low H_2O_2) and chaotic (teal, high H_2O_2) conditions. Simulations of the stress response model with gene expression noise S* do not match experimental observations unless the response model S* is coupled with the growth G and cell-interactions I model components. Therefore, noise alone cannot explain the response fluctuations. (**B**) Noisy stress response model without growth and cell-interactions components does not show expected cross-correlations: Example simulated GrxA trace shows gene expression fluctuations (left), but no cross-correlation function (CCF) peaks are seen between changes in GrxA (DGrxA) and changes in barrier cells (Dbarrier) (middle), in disagreement with experiments. Corresponding phase diagram (right) does not show the characteristic extended orbits of periodic or chaotic fluctuations. **(C)** Coupling of the growth model with the noisy response model S*+G does not generate the expected cross-correlation and phase diagram orbits, neither for low (black) nor high (teal) H_2O_2 concentrations. **(D)** Coupling of the cell-interaction model with the noisy response model S*+I generates the expected cross-correlation and phase diagram orbits, but the autocorrelation of the dynamics for low (black) and high (teal) H_2O_2 concentrations is inconsistent with experiments. **(E)** Coupling of the growth and cell interaction models with the noisy response model S*+G+I generates the expected cross-correlation and phase diagram orbits for low (black) and high (teal) H_2O_2 concentrations. (**F**) For simulated traces of the S*+I model (panel D), the ACF shows periodic oscillations even at high H_2O_2 concentration (teal), in contrast to experiments where high H_2O_2 leads to chaos and loss of ACF peaks (Fig 6C). (**G**) Correlation dimension analysis using Grassberger – Procaccia algorithm indicates that observed fluctuations are deterministic. Curves show the correlation dimension as a function of the embedding dimension from P*grxA*-SCFP3 traces of mother cells during H_2O_2 treatment at steady-state under the indicated measurement conditions. Curves for experimental traces (teal) are compared to experimental traces with randomly shuffled time points (dashed black) and to synthetic traces with data points drawn from a Gaussian distribution with the same mean and standard deviation as the experimental traces (maroon). The curves from experimental traces saturate at a finite correlation dimension of ~2 for all measurement conditions, consistent with deterministic

dynamics, whereas the curves from the randomized controls do not saturate. (970, 733 traces for cells growing in M9 glycerol and M9 glucose + 10% LB respectively, curves represent mean values with error bars representing standard deviation). (**H**) Effect of moving-mean smoothing on correlation dimension analysis. Example raw P*grxA*-SCFP3 trace (grey) and after smoothing with a filter window of 9 minutes (black dashed). (**I**) (left) Correlation dimension versus embedding dimension from P*grxA*-SCFP3 traces as in panel H for raw data (solid lines) or with moving-mean smoothing (dashed lines). (right) Application of correlation analysis to simulated GrxA traces from the full deterministic model S+G+I (dashed lines) and the full model with noisy stress response S*+G+I (solid lines).

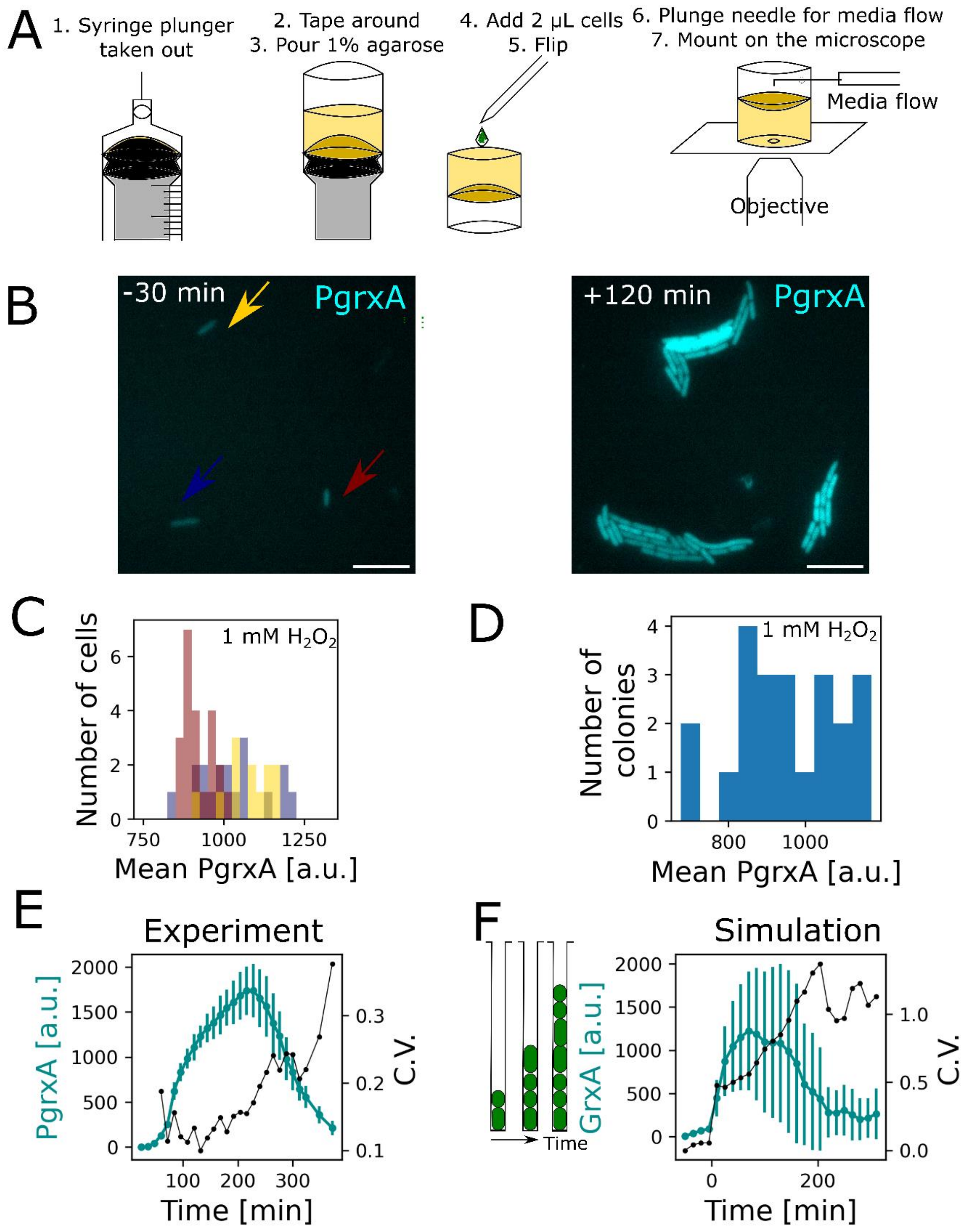

Figure S5. Bacterial colonies in experiments exhibit oxidative stress response fluctuations consistent with chaos. Related to Figure 5.

(**A**) Schematic describing the preparation of agarose pads for imaging bacterial microcolonies under continuous H_2O_2 infusion. (**B**) Snapshots of P*grxA*-SCFP3 expression of microcolonies growing from 3 single cells under 1 mM H_2O_2 treatment (left; 30 mins before treatment, right;

120 mins after treatment). (**C**) Histograms of P*grxA*-SCFP3 intensities of individual cells show heterogeneous responses at 120 minutes after start of 1 mM H_2O_2 treatment (colours corresponding to microcolonies arising from cells marked with arrows in panel B, n=54 cells). (**D**) Histograms of mean P*grxA*-SCFP3 intensities for entire colonies at 120 minutes after start of 1 mM H_2O_2 treatment showing variability in responses between different colonies (n = 22 colonies with 16 ± 12 cells per colony). (**E, F**) Oxidative stress response dynamics and cell-to-cell heterogeneity of microcolonies in experiments match model predictions. The GrxA expression (cyan lines, mean ± standard deviation across cells) initially increases upon constant 1 mM H_2O_2 treatment from t = 0 min but the average response of the whole population decreases again when a growing number of cells in the interior of the population becomes protected by the H_2O_2 scavenging activity of the cells at the edge of the colony. This divergence of the population response causes an increase over time in the coefficient of variation (C.V., black lines) of GrxA expression across cells in the colony. (**E**) Experimental results for P*grxA*-SCFP3 expression of all cells tracked in a microcolony over time (increasing from n = 2 to 737 cells over time). (**F**) Simulation of a one-dimensional population growing in a very long trench (100 µm) matches experimental results shown in panel E for microcolonies (n = 3 simulations).

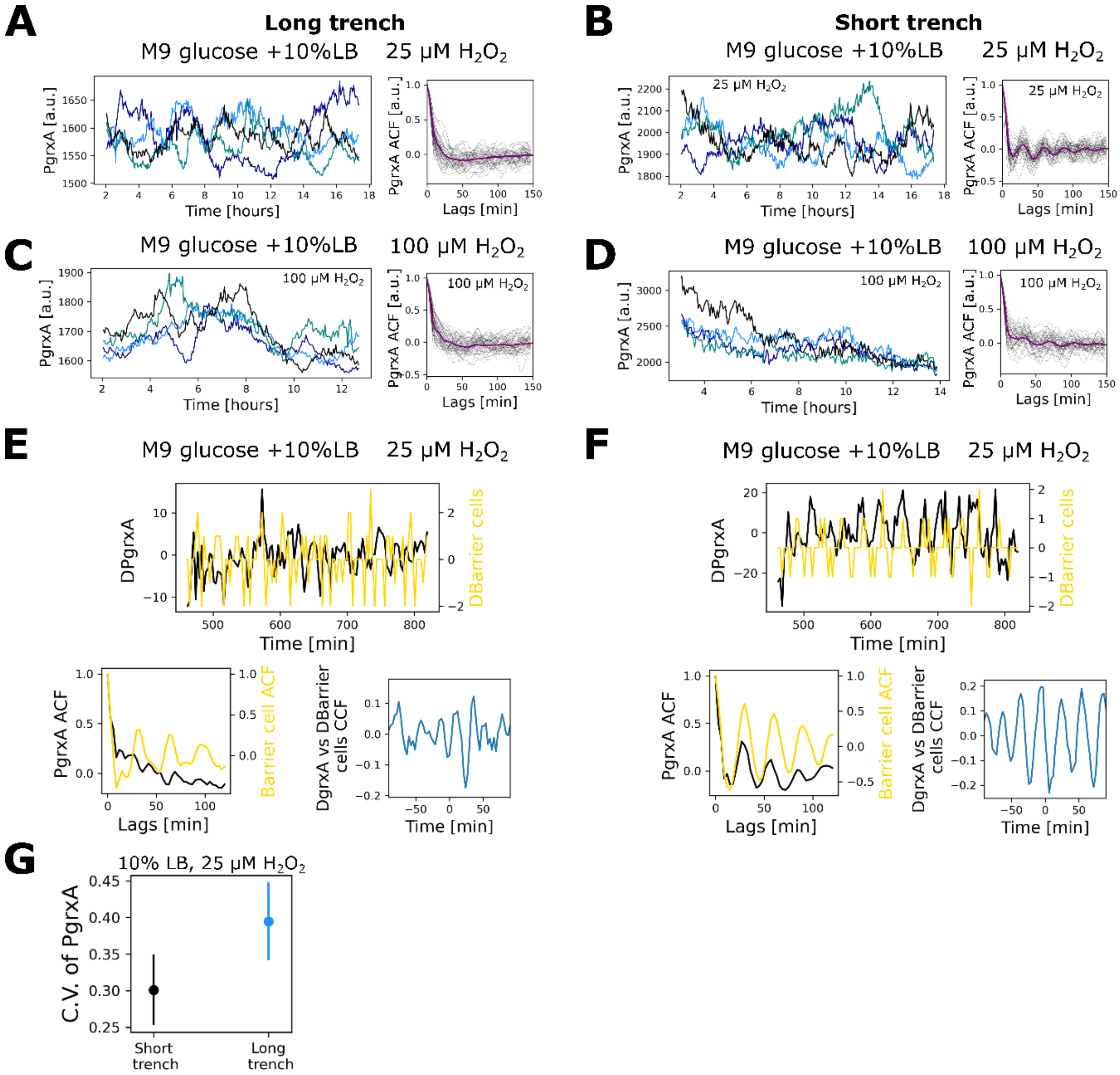

Figure S6. Deterministic chaos in oxidative stress response fluctuations at single cell level. Related to Figure 5 and 6.

Correlation analysis shows chaotic and periodic response dynamics at a single-cell level in experiments. (**A-D**) 5 representative P*grxA*-SCFP3 expression traces and ACF curves for 50 individual mother cells (black) and cell-average curve (purple). (**A**) Chaotic dynamics: M9 glucose + 10% LB with 25 μM H_2O_2 in 25 μm trenches. (**B**) Periodic dynamics: M9 glucose + 10% LB with 25 μM H_2O_2 in 10 μm growth trenches. (**C**) Chaotic dynamics: M9 glucose + 10% LB with 100 μM H_2O_2 in 25 μm trenches. (**D**) Periodic dynamics: M9 glucose + 10% LB with 100 μM H_2O_2 in 10 μm growth trenches. (**E, F**) Representative mother cell traces showing cross-correlation between changes in P*grxA*-SCFP3 expression (DpgrxA, black) and changes

in barrier cell numbers (DBarrier cells, yellow). Corresponding autocorrelation of DpgrxA (ACF, left) and cross-correlation of DpgrxA vs Dbarrier cells (CCF, right) plots for the single mother cell traces above. Cells growing in M9 glucose + 10% LB and treated with 25 μM H_2O_2 in (**E**) 25 μm trenches (chaotic dynamics) and (**F**) 10 μm trenches (periodic dynamics). (**G**) Under chaotic conditions cell-cell variability increases. Coefficient of variation (C.V.) of P*grxA*-SCFP3 expression for mother cells growing in M9 glucose + 10% LB and treated with 25 μM H_2O_2 in 25 μm (blue) and 10 μm trenches (black) (n= 3 experimental repeats, mean ± standard deviation).
