# Supplementary material for: Chaos in a bacterial stress response
Source: Curr Biol. Author manuscript; Available in PMC 2024 Oct 8. (PMC7616676; doi:10.1016/j.cub.2023.11.002)
Supplement: Supplementary Materials [file EMS199120-supplement-Supplementary_Materials.zip › 1-s2.0-S0960982223015166-mmc2.pdf]

Figure	Initial lengths of cells (in μm) at the start of the simulation (from closed to open end of trenches)	$[H_2O_2]_{external}$ (μM)	Growth rate (min^{-1})	Length of growth trench (in μm)
2A	2.14, 2.38, 3.23, 2.94	800	0.042	25
2A	2.14, 2.14, 3.24, 2.74, 3.15	800	0.042	25
2A	2.14, 2.54, 3.02, 3.02, 3.32, 3.02, 3.62	800	0.042	25
2B	2.241, 3.382, 2.831, 3.063, 3.054, 3.042, 3.751	260	0.042	25
2B	2.24101, 3.382,2.831, 3.063, 3.054, 3.042, 3.751	260	0.042	25
2B	2.24102, 3.382, 2.831, 3.063, 3.054, 3.042, 3.751	260	0.042	25
2C-D	2.241, 2.342, 3.043, 2.244, 3.067, 3.241	80	0.042	25
2C-D	2.241, 2.342, 3.043, 2.244, 3.067, 3.241	140	0.042	25
2C-D	2.241, 2.342, 3.043, 2.244, 3.067, 3.241	260	0.042	25
2E-G	3.24, 3.38, 2.83, 3.06, 3.05, 3.04, 2.56	4i for $i \in (1,101)$ and 40i for $i \in (10,101)$	0.042	25
2E-G	2.24, 2.34, 3.04, 2.24, 3.06, 3.24	4i for $i \in (1,101)$ and 40i for $i \in (10,101)$	0.042	25
2E-G	3.64, 2.54, 3.02, 3.02, 2.32, 2.62	4i for $i \in (1,101)$ and 40i for $i \in (10,101)$	0.042	25

3D	2.24, 2.34, 3.04, 2.24, 3.06, 3.24	2·i for i ∈ (1,100) Kcat = 20	0.042	25
3D	2.04, 3.18, 2.53, 2.16, 3.35, 3.74, 3.06	2·i for i ∈ (1,100) Kcat = 20	0.042	25
3D	3.24, 3.34, 3.04, 2.24, 3.06, 3.64	2·i for i ∈ (1,100); Kcat = 20	0.042	25
3E	2.24, 2.34, 3.04, 2.24, 3.06, 3.24	20,100,160	0.042	25
3F	2.24, 3.18, 2.53, 2.16, 3.35, 3.74, 3.06	162; Kcat= 0.5·i for i ∈ (1,40)	0.042	25
3G	2.24, 2.34, 3.04, 2.24, 3.06, 3.24	162, Kcat= [2.5, 9.5, 20]	0.042	25
3H	2.24, 3.18, 2.53, 2.16, 3.35, 3.74, 3.06	162, Kact=0.001·i for i ∈ (25,100)	0.042	25
3I	2.24, 2.34, 3.04, 2.24, 3.06, 3.24	162, Kact= [0.03, 0.045, 0.09]	0.042	25
4A-C	100 random initial lengths	100	0.042	25
5C	10 random initial lengths	100	0.042	25
6A, S2D-E	2.24,2.34, 3.04, 2.24, 3.06, 3.24	260	0.0105,0.021, 0.042,0.084	25
6A, S2D-E	3.24, 3.38, 2.83, 3.06, 3.05, 3.04, 2.56	260	0.0105,0.021, 0.042,0.084	25
6A, S2D-E	3.64, 2.54, 3.02, 3.02, 2.32, 2.62	260	0.0105,0.021, 0.042,0.084	25
6B, S2G-H	2.24, 2.34, 3.04, 2.24, 3.06, 3.24	260	0.042	15,20,25
6B, S2G-H	3.24, 3.38, 2.83, 3.06, 3.05, 3.04, 2.56	260	0.042	15,20,25
6B, S2G-H	3.64, 2.54, 3.02, 3.02, 2.32, 2.62	260	0.042	15,20,25
S1C	2.241, 3.382, 2.831, 3.063, 3.054, 3.042, 3.751	0	0.042	25

S1C	2.241, 3.382, 2.831, 3.063, 3.054, 3.042, 3.751	260	0.042	25
S2A	2.241, 3.382, 2.831, 3.063, 3.054, 3.042, 3.751	260	0.042	25
S2A	2.241000001, 3.382, 2.831, 3.063, 3.054, 3.042, 3.751	260	0.042	25
S2A	2.241000002, 3.382,2.831, 3.063, 3.054, 3.042, 3.751	260	0.042	25
S2B	2.241, 3.382, 2.831, 3.063, 3.054, 3.042, 3.751	260	0.042	25
S2B	2.341, 3.382, 2.831, 3.063, 3.054, 3.042, 3.751	260	0.042	25
S2B	2.44100002, 3.382, 2.831, 3.063, 3.054, 3.042, 3.751	260	0.012	25
S2C	3.24, 3.38, 2.83, 3.06, 3.05, 3.04, 2.56	400	N/A	25
S2F	3.24, 3.38, 2.83, 3.06, 3.05, 3.04, 2.56	10	0.042	N/A
S3A-D	20 random initial lengths	[12.5, 25, 37.5, 50, 62.5, 75, 87.5, 100, 500]	0.042	25
S3E-F	20 random initial lengths	12.5	0.042	25
S4A	10 random initial lengths	80, 900	0.042	25
S4B	10 random initial lengths	10	N/A	N/A
S4C	10 random initial lengths	10, 40	0.042	N/A
S4D, F	10 random initial lengths	80, 900	N/A	25
S4E	10 random initial lengths	80, 900	0.042	25

S4I	10 random initial lengths	100	0.042	25
S5F	3.24, 3.38, 2.83, 3.06, 3.05, 3.04, 2.56	1000	0.042	100
S5F	2.24, 2.34, 3.04, 2.24, 3.06, 3.24, 2.78	1000	0.042	100
S5F	3.64, 2.54, 3.02, 3.02, 2.32, 2.62, 2.32, 3.15	1000	0.042	100
