# Supplementary material for: Chaos in a bacterial stress response
Source: Curr Biol. Author manuscript; Available in PMC 2024 Oct 8. (PMC7616676; doi:10.1016/j.cub.2023.11.002)
Supplement: Supplementary Materials [file EMS199120-supplement-Supplementary_Materials.zip › 1-s2.0-S0960982223015166-mmc9.pdf]

Current Biology

Article

Chaos in a bacterial stress response

Graphical abstract

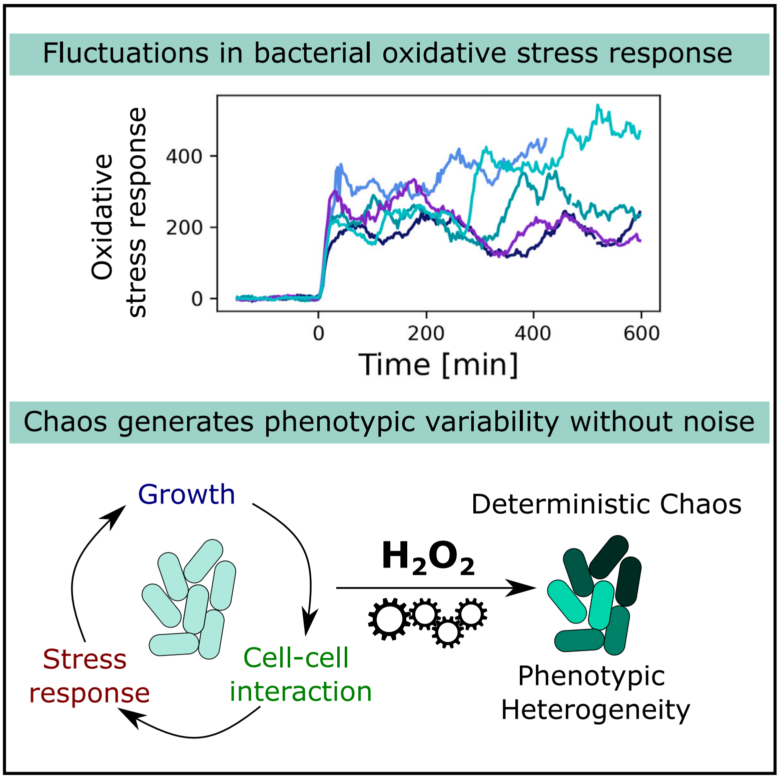

Highlights

- Chaos is seen in the *E. coli* response to oxidative stress
- Feedback between gene regulation, growth rate, and cell-cell interactions causes chaos
- Modeling, analysis, and perturbation experiments rigorously demonstrate chaos
- Chaos generates phenotypic variability without noise

Authors

Divya Choudhary, Kevin R. Foster, Stephan Uphoff

Correspondence

kevin.foster@biology.ox.ac.uk (K.R.F.),
stephan.uphoff@bioch.ox.ac.uk (S.U.)

In brief

Chaotic processes are well understood in the physical sciences to generate seemingly random dynamics from purely deterministic mechanisms. The existence and role of chaos in biological systems are less certain. Here, Choudhary et al. show that chaos explains the variability in the responses of individual bacterial cells to oxidative stress.

Choudhary et al., 2023, Current Biology *33*, 5404–5414
December 18, 2023 © 2023 The Author(s). Published by Elsevier Inc.
https://doi.org/10.1016/j.cub.2023.11.002

CellPress

CellPress
OPEN ACCESS

Current Biology

Article

Chaos in a bacterial stress response

Divya Choudhary,[1,4] **Kevin R. Foster,**[1,2,*] **and Stephan Uphoff**[1,3,5,*]
[1]Department of Biochemistry, University of Oxford, Oxford OX1 3QU, UK
[2]Department of Biology, University of Oxford, Oxford OX1 3SZ, UK
[3]X (formerly Twitter): @stephan_uphoff
[4]X (formerly Twitter): @Divya_ch_
[5]Lead contact
*Correspondence: kevin.foster@biology.ox.ac.uk (K.R.F.), stephan.uphoff@bioch.ox.ac.uk (S.U.)
https://doi.org/10.1016/j.cub.2023.11.002

SUMMARY

Cellular responses to environmental changes are often highly heterogeneous and exhibit seemingly random dynamics. The astonishing insight of chaos theory is that such unpredictable patterns can, in principle, arise without the need for any random processes, i.e., purely deterministically without noise. However, while chaos is well understood in mathematics and physics, its role in cell biology remains unclear because the complexity and noisiness of biological systems make testing difficult. Here, we show that chaos explains the heterogeneous response of *Escherichia coli* cells to oxidative stress. We developed a theoretical model of the gene expression dynamics and demonstrate that chaotic behavior arises from rapid molecular feedbacks that are coupled with cell growth dynamics and cell-cell interactions. Based on theoretical predictions, we then designed single-cell experiments to show we can shift gene expression from periodic oscillations to chaos on demand. Our work suggests that chaotic gene regulation can be employed by cell populations to generate strong and variable responses to changing environments.

INTRODUCTION

The birth of chaos theory was highly significant because it made clear that unpredictable patterns in nature can arise without stochasticity, i.e., purely deterministically.[1] Many seemingly noisy systems were subject to reanalysis and reinterpreted as chaotic rather than stochastic, including examples from biology such as ecological dynamics,[2–7] gene expression,[8,9] immune system dynamics,[10,11] neural signal dynamics,[12–15] circadian rhythms,[16,17] and heart beats.[18] However, the underlying causes of dynamics in biological systems are not as well understood as in the physical or chemical sciences.[19,20] As a result, the inference of chaos in biology often rests upon mathematical models alone,[3,4,12,21–24] which is not sufficient to demonstrate that chaos actually occurs in the biological system itself.

Empirically, chaotic dynamics have been inferred in biological data by statistical detection tools.[2,13,25,26] However, it is challenging to distinguish chaotic from stochastic causes in this manner because the inference methods are highly sensitive to measurement noise and random fluctuations that are inherent to all biological processes.[27] This problem was recently illustrated by Toker et al., who applied new analysis tools to both physical and biological systems.[28] Although they found evidence for chaos in physical and simulated biological data, they concluded that measured heart rate data are stochastic, in spite of a large number of papers having previously concluded that they are chaotic.[18] Chaos, then, has proved much more difficult to evidence in biological systems than in physical ones, leaving the importance of chaotic dynamics for biology in doubt.

In response to environmental threats, bacteria have evolved stress responses that drive rapid physiological adaptation.[29] Stress responses are intensively studied because they are central to the ways in which bacteria survive environmental threats and diverse treatments, including antibiotics.[30–33] We serendipitously discovered chaotic behavior in a theoretical model of one of the major bacterial stress responses, specifically the oxidative stress response of the model species *E. coli*. This discovery allowed us to leverage the detailed understanding and tractability of *E. coli* to overcome the typical challenges faced when studying chaos in biological systems. Under high hydrogen peroxide (H_2O_2) stress, a cell will strongly induce the expression of proteins that remove H_2O_2 within the cell and thereby lower the concentration of H_2O_2 in its vicinity. Our work shows that this response, in combination with the responses of surrounding cells, perturbs the regular periodicity of cell growth, driving the stress response dynamics from periodic oscillations to chaotic fluctuations. By identifying the drivers of chaos, we are able to predict the conditions when it will be present and when it will be lost, and we validate these predictions empirically. In this way, we provide clear experimental evidence of chaos in a living system. Our results further suggest that chaotic gene regulation could be common in the bacterial responses to diverse types of stresses and offer functional benefits.

RESULTS

Chaos is predicted in a bacterial stress response

Genetically identical bacteria often display considerable cell-to-cell variability in their responses to the environment[34] (Figure 1A). Stress responses can be particularly variable,[35] and it is often assumed that this variability results from stochastic processes inside the cell. Noisy as well as oscillatory response patterns have been observed in bacteria exposed to reactive oxygen

 © 2023 The Author(s). Published by Elsevier Inc.
This is an open access article under the CC BY license (http://creativecommons.org/licenses/by/4.0/).

CellPress
OPEN ACCESS

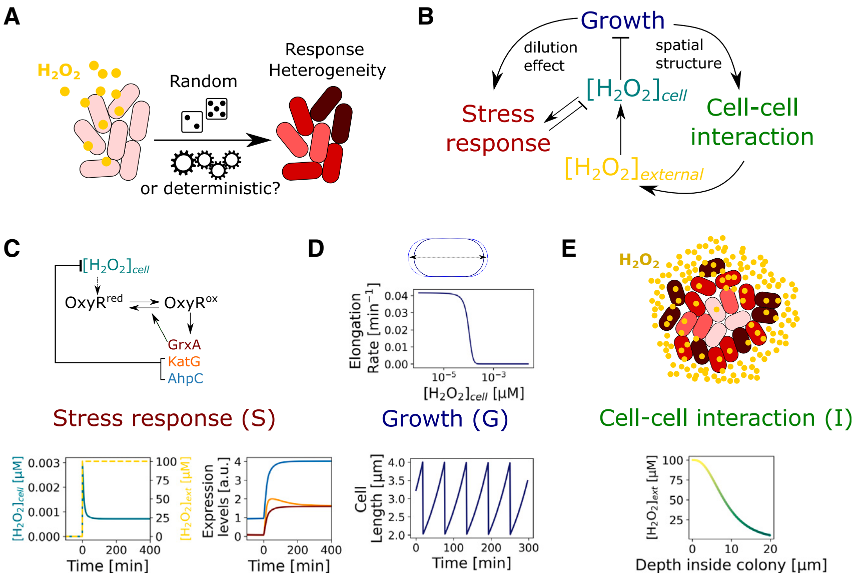

Figure 1. Modeling the oxidative stress response in bacterial populations

(A) Environmental stress, such as H_2O_2 exposure, induces heterogeneous responses in bacterial populations, which could be caused by stochastic or deterministic mechanisms.

(B) H_2O_2 affects cell growth rates (G) and triggers an intracellular stress response (S) that creates stressor gradients by cell-cell interactions (I). S-G-I feedback modulates H_2O_2 concentration in space and time, both outside and inside the bacteria ($[H_2O_2]_{external}$, $[H_2O_2]_{cell}$, respectively).

(C) Schematic of the core OxyR gene regulatory circuit corresponding to the stress response component of the model. It predicts the expression dynamics of proteins that scavenge intracellular H_2O_2 (KatG, AhpCF) and control OxyR oxidation status (GrxA). Model output illustrated for constant $[H_2O_2]_{external}$ exposure from 0 min.

(D) The growth model describes the inhibition of cell elongation by H_2O_2. In turn, the growth dynamics feed into the stress response model by determining the dilution rate of enzymes. Top: cell elongation rate as a function of intracellular H_2O_2. Bottom: exponential growth and division cycles of a single cell without H_2O_2 treatment.

(E) Cell-cell interactions are described by a reaction-diffusion model where intracellular scavenging of H_2O_2 creates a stress gradient from the edge to the interior of a cell population. Changes in the number and arrangement of cells in the population are determined by the growth model. See also Figure S1.

and nitrogen species.[36–39] Such behavior could arise when the induction of detoxifying enzymes reduces the local concentration of stressor molecules,[40] leading to dynamic feedbacks. Indeed, recent work found that cellular heterogeneity in the response of *E. coli* to oxidative stress by hydrogen peroxide (H_2O_2) is driven by cell-cell interactions rather than intracellular noise,[41] where the response of each cell is determined by the hydrogen peroxide scavenging activity of its neighbors. This project began with the goal of better understanding the impact of this response on cell-to-cell variability via a numerical model (Figures 1 and S1). The model captures three coupled processes (Figures 1B and S1). First is the dynamics of the intracellular stress response (named S in the model). In *E. coli*, these occur when H_2O_2 oxidizes the transcription factor OxyR, which induces expression of H_2O_2-scavenging enzymes AhpCF and KatG, and the glutaredoxin-1 GrxA that converts oxidized OxyR back to its reduced form[33,42] (Figure 1C). Second is the inhibitory effect of H_2O_2 on the growth rate of the cells (named G in the model) (Figure 1D) and, finally, there is the impact of cell-cell interactions (named I in the model) (Figure 1E). These interactions arise because the uptake of H_2O_2 by one cell can lower the concentration of H_2O_2 for surrounding cells.

We began by solving a one-dimensional (1D) version of the model, where cells are exposed to a constant external H_2O_2 concentration from one direction (Figure S1; Video S1). This simple geometry allowed us to explore the behavior of the system at steady state and is highly amenable to empirical testing via microfluidic growth trenches (using a device called the "mother machine"), which are commonly used in experiments with bacteria.[41,43] We then followed the stress response (GrxA expression level) in so-called "mother cells" located at the base of the cell group farthest from the source of treatment; the other cells are termed "barrier cells" (Figure 2A; Video S1). Given that the model is purely deterministic, with no noise terms, we were surprised to observe seemingly random fluctuations in the stress response of individual mother cells (Figure 2A; Video S1). Moreover, repeated runs of the model yielded highly variable stress response trajectories, which we initially found confusing because model parameters were identical throughout. The only source of variability was that each run of the model began with unsynchronized cells, i.e., at random points in the cell cycles. To explore whether this was the source of the variation, we ran the model with synchronized barrier cells but shifted the initial cell cycle progression of only the mother cell by $2.5 \cdot 10^{-4}$% and $5 \cdot 10^{-4}$%. The resulting three trajectories were initially indistinguishable but began to diverge significantly after ~6 h post treatment with H_2O_2(Figures 2B, S2A, and S2B; Video S1 [bottom]). Once diverged, the dynamics became completely different for the three model runs. This extreme sensitivity to small differences in initial conditions of a deterministic model suggests chaotic dynamics.[28,44]

We next visualized response trajectories as phase diagrams across a range of H_2O_2 concentrations. These show the defined and closed orbits of periodic oscillations for lower H_2O_2 concentrations, but at higher H_2O_2, we observed the dense aperiodic orbits that are indicative of chaotic fluctuations (Figures 2C and 2D). These fluctuations can also be seen in a bifurcation diagram, which shows the extrema values of the fluctuations for cells as a function of H_2O_2 concentration. The form of the resulting diagram is characteristic of a chaotic system that shifts from periodic to chaotic regimes as H_2O_2 concentration increases (Figure 2E). To test formally for chaotic dynamics, we computed the Lyapunov exponent λ, which is positive for a chaotic system where a small perturbation in initial conditions leads to exponential divergence of the trajectories. As expected from the bifurcation diagram, cells in the model at higher H_2O_2 concentrations showed chaos ($\lambda > 0$), while lower concentrations caused predominantly periodic oscillations in responses ($\lambda < 0$) (Figure 2F).

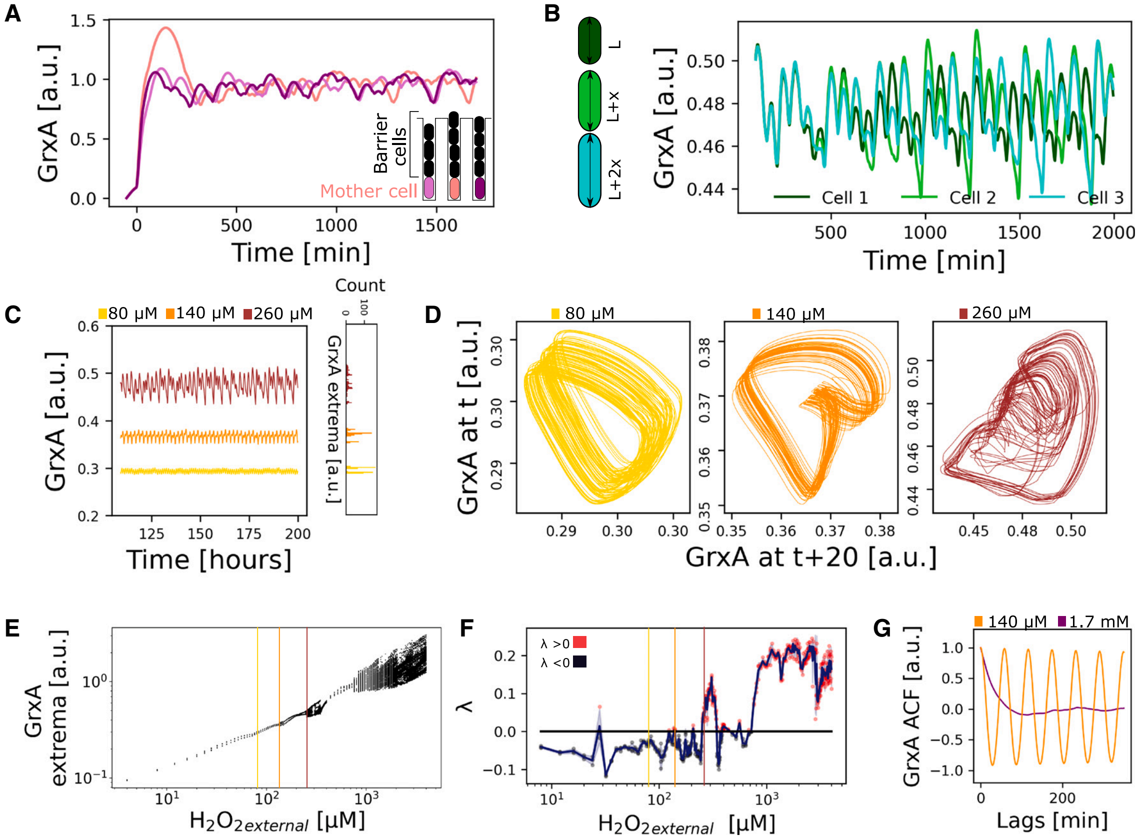

Figure 2. The model predicts chaos in the stress response

(A) Oxidative stress response fluctuations in individual "mother cells" at the base of a one-dimensional population with "barrier cells" positioned closer to the H_2O_2 source. The model produces seemingly random dynamics of GrxA protein expression level during continuous H_2O_2 treatment from t = 0 min. The curves represent three independent simulation runs, starting with unsynchronized cells at random points in the cell cycles.

(B) Stress response fluctuations diverge greatly over time, even if the differences in initial conditions are very small: here shown by the GrxA dynamics for 3 mother cells that differ very slightly in their initial stage of the cell cycle ($2.5 \cdot 10^{-4}$% and $5 \cdot 10^{-4}$ % length differences).

(C) (Left) Representative GrxA dynamics for a mother cell with continuous treatment at different H_2O_2 concentrations (80, 140, and 260 μM). (Right) Histogram of counts of extrema detected for 3 mother cells for different H_2O_2 concentrations.

(D) Phase diagrams for the GrxA dynamics of the mother cells presented in (C), displaying bistable (80 μM) and multistable periodic oscillations (140 μM) and chaotic fluctuations (260 μM).

(E) Bifurcation plot of the GrxA extrema values over a range of H_2O_2 concentrations (n = 3 simulations per concentration). Vertical lines represent example traces in (C) and (D).

(F) A positive Lyapunov exponent (λ) shows chaotic divergence from initial conditions, computed for GrxA dynamics at different H_2O_2 concentrations. Individual points represent single mother cells, with red dots for chaos ($\lambda > 0$) and black dots for periodicity ($\lambda \leq 0$). Blue line and shaded region show mean ± SD of n = 3 cells simulated per H_2O_2 concentration.

(G) The autocorrelation function (ACF) distinguishes periodic and chaotic response fluctuations. Mean of ACF for GrxA of mother cells decreases steeply for chaotic traces under high H_2O_2 treatment (1.7 mM, purple) and shows regular peaks for periodic traces under low H_2O_2 treatment (140 μM, orange) (n = 3 simulations).

See also Figure S2 and Video S1.

Note that the frequent and abrupt transitions between periodic and chaotic behavior that we observed in our simulations are typical even for the simplest mathematical models of chaos.[1] An autocorrelation curve of the aperiodic traces also decreased quickly over time, again indicative of chaos, whereas periodic oscillations showed characteristic autocorrelation peaks (Figure 2G).

To explore the generality of our observations, we replaced the detailed model of the oxidative stress response with a simpler version for a generic stress response (Figure 3A). Here, cells take up toxic molecules from their surroundings and protect themselves by producing enzymes that reduce intracellular toxin concentrations (Figures 3B and 3C). Similar to the oxidative stress model, we observed that an increased toxin concentration leads to chaotic enzyme expression dynamics (Figures 3D and 3E). Furthermore, the system becomes more prone to chaotic behavior as the catalytic efficiency of the enzyme or its expression rate increase (Figures 3F–3I).

Observational data also suggest chaos

We next performed experiments on *E. coli* populations growing in mother machine chips under constant H_2O_2 treatment, where

CellPress
OPEN ACCESS

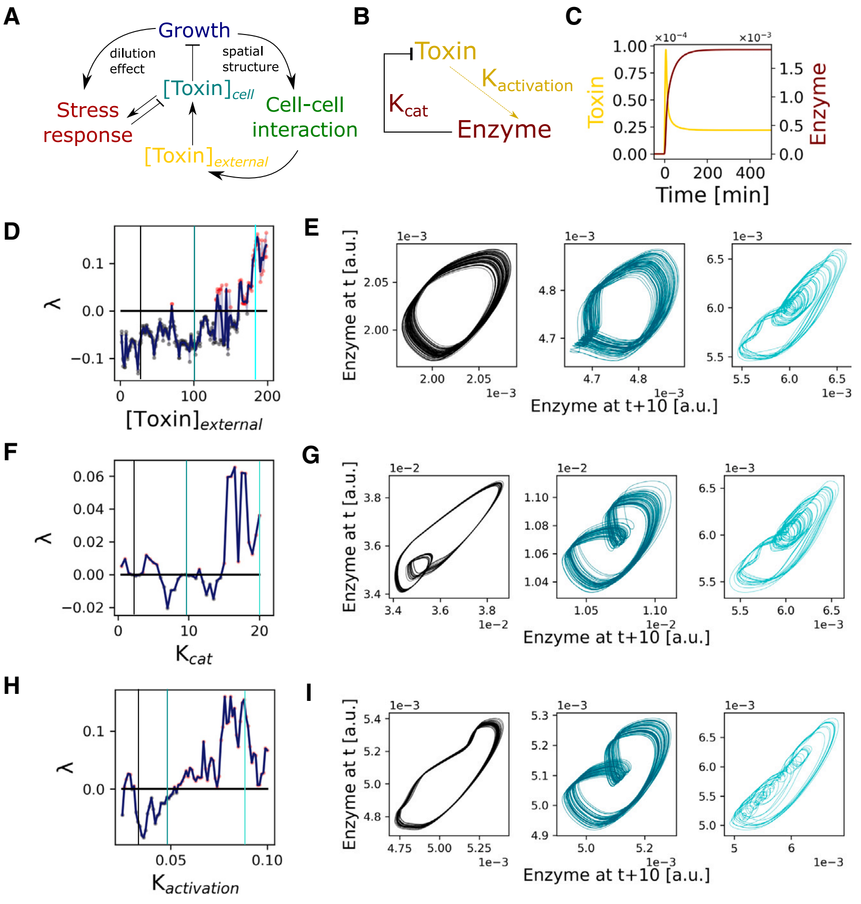

Figure 3. Chaos emerges in a general model of stress responses in cell populations

(A) Uptake of toxins reduces cell growth rates (G) and triggers an intracellular stress response (S) that creates toxin gradients by cell-cell interactions (I). S-G-I feedback modulates toxin concentration in space and time, both outside and inside the bacteria ($[Toxin]_{external}$, $[Toxin]_{cell}$ respectively).

(B) Schematic of a generic stress response in which exposure to a toxin induces the expression of a detoxifying enzyme with rate $K_{activation}$ that removes toxin with rate K_{cat}.

(C) Model output illustrates the expression dynamics of the enzyme (maroon) and the intracellular toxin concentration (yellow) for constant external toxin exposure from t = 0 min without S-G-I feedback.

(D) A positive Lyapunov exponent (λ) shows chaotic divergence from initial conditions, computed for enzyme expression dynamics over a range of toxin concentrations (n = 3 simulations per toxin concentration). Higher external toxin concentrations lead to chaos.

(E) Phase diagrams for the enzyme expression dynamics of a mother cell at toxin concentrations marked by vertical lines in (D), displaying periodic oscillations and chaotic fluctuations.

(F) Higher K_{cat} of the enzyme increases chaotic behavior. Lyapunov exponent for enzyme expression dynamics over a range of K_{cat} values.

(G) Phase diagrams for the enzyme expression dynamics of a mother cell at K_{cat} values marked in (F), displaying periodic oscillations and chaotic fluctuations.

(H) Higher expression rate $K_{activation}$ of the enzyme increases chaotic behavior. Lyapunov exponent for enzyme expression dynamics over a range of $K_{activation}$ values.

(I) Phase diagrams for the enzyme expression dynamics of a mother cell at $K_{activation}$ values marked in (H), displaying periodic oscillations and chaotic fluctuations.

the stress response level is measured with a transcriptional P*grxA*-SCFP3 gene expression reporter and time-lapse fluorescence microscopy. We first tested the ability of the model to predict the general characteristics of the stress response. Adjusting model parameters to the measurement conditions (Figures S3A–S3D) demonstrated an excellent quantitative agreement between our theory and the experiments (Figure 4A; Video S2). Both showed similar spatio-temporal response dynamics and the same gradient in stress response level along the row of cells (Figure 4A; Video S2). Experiments also matched the theoretical prediction that sudden H_2O_2 treatment triggers an induction of stress response, which coincides with a transient dip in the cell elongation rate followed by adaptation (Figures 4B, 4C, S3E, and S3F). Importantly, like the model, the mother cells in experiments displayed large fluctuations in stress response level during constant H_2O_2 treatment (Figure 4D). The autocorrelation curves from these traces decreased quickly over time, indicating a lack of periodicity in the dynamics, consistent with chaotic behavior (Figure 4E).

We further applied the "chaos decision tree algorithm" of Toker et al.,[28] which is an analysis pipeline that uses the permutation entropy to categorize dynamics as stochastic or deterministic. The pipeline classified 92% of the measured stress response trajectories as deterministic (Figures 5A, 5B, and S3M–S3Q), supporting the prediction of the model that the fluctuations are predominantly a consequence of deterministic chaos and not caused by noise. Most of the 8% of trajectories that were categorized as stochastic corresponded to cells that had died at the onset of H_2O_2 treatment (Figures 5A, 5B, and S3M–S3Q). Active growth dynamics are hence required for chaotic response behavior.

As another test to distinguish deterministic chaos from noise, we applied the Grassberger-Procaccia algorithm.[45] This algorithm identifies the correlation dimension (or fractal dimension), which should be low when the response fluctuations are driven by a deterministic process with a small number of effective variables, but tends to infinity for a truly stochastic process.[46] We found a finite correlation dimension of ~2 for the GrxA dynamics in experiments and simulations, indicating determinism in the system (Figure 5C). The analysis, therefore, is consistent with our model prediction that deterministic response fluctuations are generated by a simple cyclic cell growth pattern that generates oscillations in the number of barrier cells. Indeed, GrxA dynamics were negatively correlated with changes in the number of barrier cells in experiments, consistent with the expectation that an increase in the number of barrier cells reduces the local H_2O_2 concentration and thus the stress response level (Figure S4A).

The use of the mother machine allowed us to follow cell trajectories over long periods, which is important for our ability to test

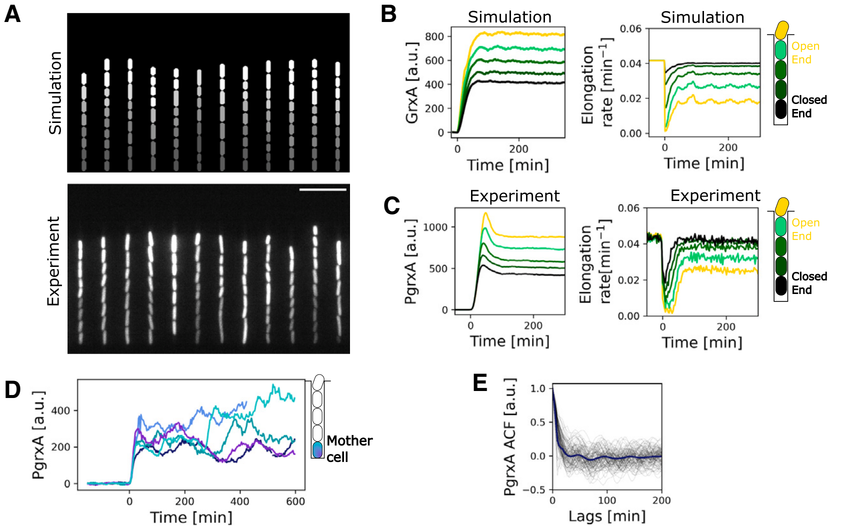

Figure 4. Experiments on the oxidative stress response in *E. coli* reveal a good fit with the modeling predictions

(A) Top: model simulation snapshot of GrxA expression after 90 min of 100 μM H_2O_2 treatment. Bottom: snapshot of experiment with *E. coli* cells growing in a "mother machine" expressing P*grxA*-SCFP3 after 90 min of 100 μM H_2O_2 treatment. Scale bar, 10 μm.

(B and C) Model simulation predictions and experimental data for mean GrxA expression (left) and mean cell elongation rates (right) under constant 100 μM H_2O_2 treatment from t = 0 min for cells at different positions in growth trench (n = 100 simulated trenches and 3 experimental repeats).

(D) P*grxA*-SCFP3 dynamics of individual cells diverge greatly over time under constant 100 μM H_2O_2 treatment from t = 0 min in experiments (5 representative mother cells shown).

(E) The steep decay of the autocorrelation function (ACF) of the response fluctuations is consistent with chaos. Mean of ACF for P*grxA*-SCFP3 of mother cells with 100 μM H_2O_2 treatment (blue, 3 experimental repeats). ACF for individual cell traces shown in black (n = 100).

See also Figure S3 and Video S2.

the model's predictions. However, the 1D structure introduced by the mother machine is also potentially unrepresentative of the way that bacteria normally grow and respond to stresses. To study cells in a more realistic setting, we imaged two-dimensional (2D) microcolonies with continuous H_2O_2 treatment (Figures 5D, S5A, and S5B; Video S3). Although these experiments cannot monitor cells over a long time, we again observed substantial heterogeneity in responses between individual cells, both within and across microcolonies, which is consistent with chaotic behavior (Figures 5E, S5C, and S5D). Moreover, as the colony grows and expands, the stress response eventually decreases and becomes more uniform (Figure S5E). This behavior is predicted by the model: scavenging reduces the H_2O_2 concentration in a larger colony and, with this, the potential for chaotic responses is predicted to decrease (Figure S5F).

Experimental tests make or break chaos

Our model predicts the existence of chaotic behavior in a biological system—the oxidative stress response of *E. coli*—and our observational data are consistent with chaos. Together, these two approaches lend support for chaotic behavior and they reflect the typical standard of evidence in biological systems, where modeling predicts chaos and/or observation of seemingly chaotic dynamics are reported. However, there are problems with such evidence. Most obviously, a modeling prediction is just that; it does not demonstrate that chaos actually occurs in a biological system. Second, tests for chaos from observational data are challenging when the underlying causes of the dynamics are uncertain.

We, therefore, sought to leverage the tractability of our study system to provide strong evidence of chaos in a biological system via targeted perturbation. In particular, if the dynamics are indeed deterministic, then it should be possible to shift the responses away from the chaotic regime into the parameter space where periodic oscillations occur, whereas this should not be possible for stochastic fluctuations (e.g., caused by gene expression noise). To evaluate this prediction, we returned to our model to identify changes that remove the chaos from the dynamics and shift them to periodic oscillations. This analysis revealed that feedback between each of the three components of the model—cell growth (G), cell interactions (I), and stress response (S) (Figure 1B)—is required for the emergence of chaotic dynamics (Figures S2C and S2F). Specifically, S is always required as it is the core of the stress response. Without the cell-cell interaction component of the model, no fluctuations were observed for the GrxA traces (Figure S2C). When the cell growth component was uncoupled from the model, i.e., the growth rate was unaffected by the H_2O_2 treatment, then the response oscillations were no longer chaotic but periodic (Figure S2F).

From here, we identified parameter changes for each component of the model that are predicted to shift stress response dynamics from chaotic to periodic. These changes were as follows: (1) reduce cell growth rate to slow down the oscillations in the number of cells per trench (G) (Figures 6A and S2F-S2H), (2) reduce cell numbers to lower the effects of cell-cell interaction (I) (Figures 6B and S2C–S2E), and (3) reduce the stress response (S) (Figures 2 and 6C). For each case, we then devised a way to make this manipulation experimentally (Figures 6A–6C and S3G–S3L): (1) growth rates were reduced by switching to a less-favored carbon source for *E. coli* (from glucose to glycerol) (Figure 6A; Video S4), (2) the protective effect of cell-cell interactions was reduced by manufacturing a modified mother machine with fewer cells in each growth channel (Figure 6B; Video S5), and (3) the strength of the stress response was reduced by lowering the concentration of H_2O_2 (Figure 6C; Video S6).

In each case, we followed the dynamics of the stress response in mother cells as before and used autocorrelation analysis to test for chaos. If the dynamics are periodic, one will see a characteristic autocorrelation that peaks at the frequency of the periodicity in the data. If the dynamics are chaotic, by contrast, no such peak in the autocorrelation is seen. However, if the fluctuations are stochastic, then we should not observe autocorrelation peaks under any condition. As expected, applying this

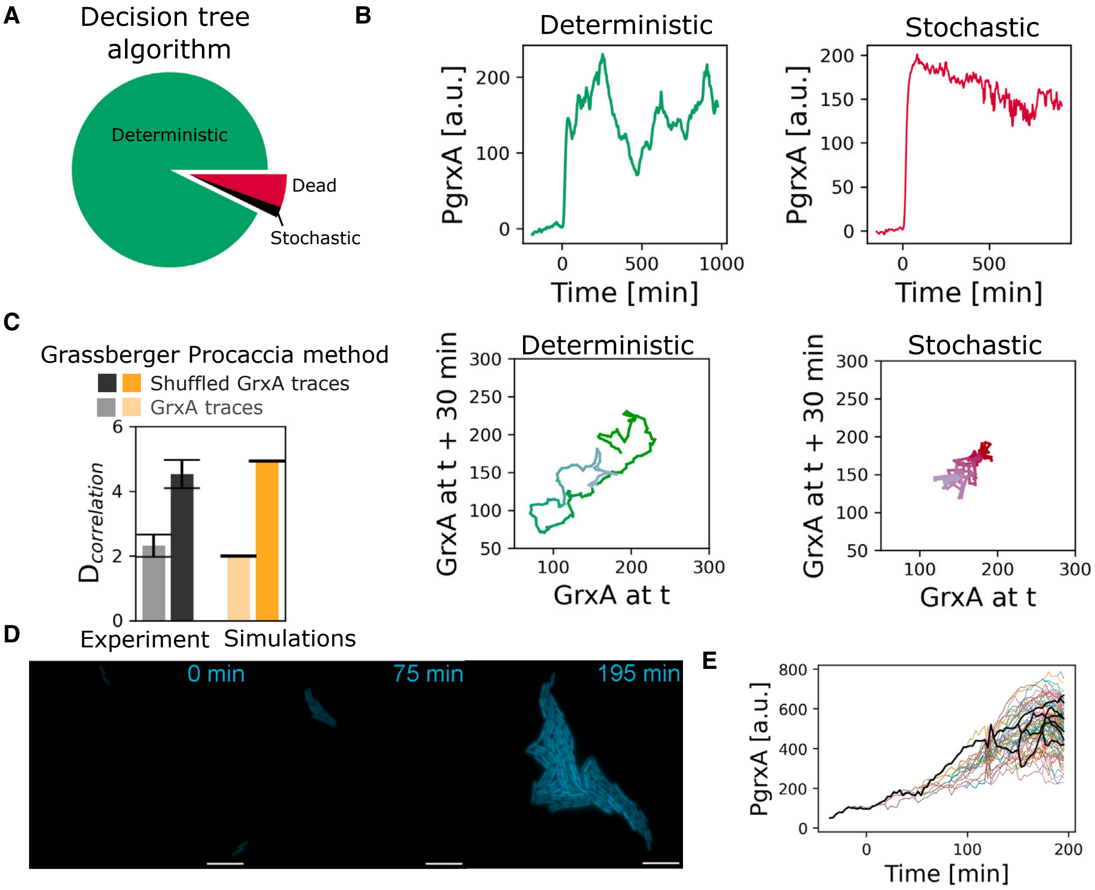

Figure 5. Measurements of stress response dynamics in *E. coli* are consistent with chaos

(A) Decision tree algorithm by Toker et al.[28] suggests that most mother cells in experiments display deterministic and chaotic response dynamics under 100 μM H_2O_2 treatment. Pie-chart indicates the fraction of dead (red) and alive (green/black) mother cells detected as having stochastic (black/red) or deterministic (green) dynamics under 100 μM H_2O_2 (n = 3,581 cells, 3 experimental repeats).

(B) P*grxA* traces (top) and their phase diagrams (bottom) for representative mother cell traces treated with 100 μM H_2O_2 treatment from t = 0 min, which are classified as deterministic (green) or stochastic (red).

(C) Bar plots show mean and standard deviation of maximal correlation dimension for experimental (black) and model (orange) GrxA traces of mother cell with (dark) or without shuffling (light) under 100 μM H_2O_2 treatment, as computed by the Grassberger-Procaccia method. Random shuffling was performed as a control to remove temporal relation between data points. The low correlation dimension is consistent with determinism in experiments and simulations.

(D) Stress response dynamics of cells growing in a colony are consistent with chaos. Snapshots of P*grxA*-SCFP3 expression with 1 mM H_2O_2 treatment from t = 0 min show cell-cell variability (scale bar, 10 μm).

(E) Single-cell trajectories from the colony experiment in (D) are consistent with chaotic divergence of stress response dynamics. See also Figures S3, S4, S5, and S6 and Video S3.

test revealed no peak in the autocorrelation function for experimental conditions that yield chaos (cyan traces in Figures 6A–6C, S6A, S6C, and S6E). By contrast, in all three cases designed to remove chaos, we observe peaks in the autocorrelation function (black traces in Figures 6A–6C, S6B, S6D, and S6F).

In summary, we were able to identify three conditions that break the chaos in the model. We then demonstrate that making these manipulations in experiments also shifts cell responses away from chaotic to periodic oscillations.

A simple deterministic process explains chaotic oxidative stress response fluctuations

The above analyses all strongly support a deterministic origin to the response dynamics. However, this does not imply the responses are entirely devoid of any noise. The number of OxyR molecules and H_2O_2 scavenging enzymes per cell is expected to fluctuate randomly due to gene expression noise.[47] Might these fluctuations still be important for the response dynamics when combined with the deterministic causes? To investigate this, we added gene expression noise to our model by introducing a stochastic term in each molecular component of the stress response model (S*) and coupled it with the cell growth or cell interaction model components as before (Figure S4). Although the resulting response dynamics superficially matched aspects of the experimental data, they disagreed with key features. Specifically, none of the noisy response models (S*, S*+I, S*+G) showed both (1) loss of autocorrelation peaks for higher H_2O_2 concentrations and (2) negative cross-correlation between response fluctuations and the number of cells per trench. These findings again support our conclusion that the

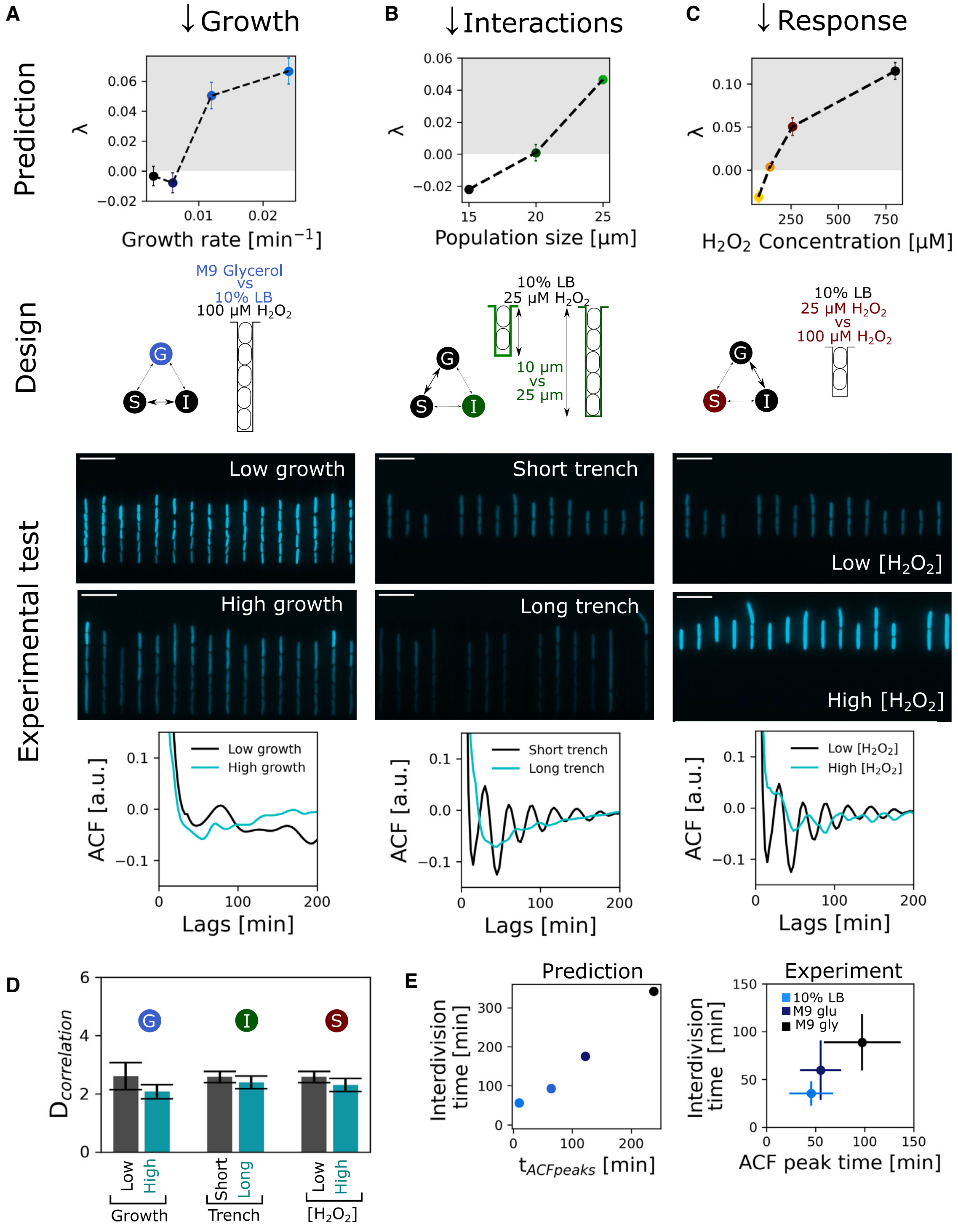

Figure 6. Predicted perturbations make or break chaos in experiments

Model predicts that chaos no longer occurs for reduced strength of either of the model components (growth, G; interactions, I; or response, S). Mean and standard deviation of Lyapunov exponent (λ) show a transition from deterministic ($\lambda \leq 0$) to chaotic ($\lambda > 0$) GrxA dynamics in simulations of cells with increasing (A) growth rates, (B) population size, and (C) H_2O_2 concentration. Experimental designs to test model predictions by changing (A) growth media, (B) trench lengths, and (C) H_2O_2 concentrations. Snapshots (scale bars, 10 µm) of P*grxA*-SCFP3 90 min after start of treatment for cells growing in (A) M9 glycerol (slow growth, top) or M9 glucose + 10% LB (fast growth, bottom) in 25-µm trenches treated with 100 µM H_2O_2.

(B) M9 glucose + 10% LB in 10-µm (2–4 cells per trench, top) or 25-µm (5–7 cells per trench, bottom) trenches treated with 25 µM H_2O_2.

(C) M9 glucose + 10% LB in 10-µm trenches treated with 25 µM H_2O_2 (top) or 100 µM H_2O_2 (bottom). Autocorrelation analysis demonstrates the predicted transitions from periodic to chaotic dynamics in experiments. ACF curves of P*grxA*-SCFP3 dynamics show characteristic peaks for periodic oscillations (black);

(legend continued on next page)

fluctuations are predominantly deterministic. Only when we coupled the noisy response with the full model of growth and cell interactions (S*+G+I) did we recover the dynamics seen in experiments, but this was already the case for the model without noise. Therefore, the model can tolerate the addition of noise but does not require it for the generation of unpredictable response fluctuations.

We find further evidence of the importance of deterministic processes over stochastic ones when we apply the Grassberger-Procaccia algorithm to the noisy response models. In all cases, the noisy models do not converge on a low correlation dimension, whereas the deterministic model had a correlation dimension of ~2 (Figures S4G–S4I). This means that although the model includes multiple dynamic variables for each of the interacting cells in a trench, the response dynamics of one focal cell are actually driven by a deterministic process with only ~2 effective variables. Strikingly, the experimental data had the same effective correlation dimension of ~2 across all growth conditions used in our tests for chaos (varying growth rate, trench length, H_2O_2 concentration), irrespective of whether the dynamics were periodic or chaotic (Figures 6D and S4G–S4I). This finding suggests that one simple deterministic process is responsible for generating all the observed response dynamics in experiments, from periodic to chaotic. Moreover, both our model and experiments suggest the cause of this determinism is strongly linked to the cell cycle (Figures S2F–S2H and S4A). Indeed, the timing of the peaks in the autocorrelation curves matches the cell cycle duration in each experimental condition, which is again predicted by the model (Figure 6E).

How does one process generate periodic oscillations under some conditions and chaotic fluctuations under others? Periodic oscillations occur at low stress levels when the cell growth rate is constant. Here, the number of cells in a given locality oscillates at regular intervals as cells grow and divide, leading to periodic changes in the H_2O_2 concentration around each cell. At higher stress treatments, the growth rate of each cell becomes sensitive to the H_2O_2 concentration. The cells then grow at variable rates and their local numbers change without a fixed period, leading to irregular oscillations in H_2O_2. In this way, the stress response dynamics transition to chaos.

DISCUSSION

Our work has identified chaos in a bacterial stress response. This finding shows that seemingly random phenotypic heterogeneity can be generated by deterministic rather than stochastic processes. That is, regulatory circuits are able to generate unpredictable outputs, even when the underlying mechanisms are entirely deterministic. Such cases are called chaotic because they have the property of amplifying infinitesimally small differences in the initial conditions to an extent that forecasting the long-term behavior is impossible—no matter how accurately the initial conditions can be defined. Although noise due to molecular fluctuations is certainly present in cells, our model and experiments show that phenotypic heterogeneity among cells can arise without the requirement for stochasticity. Moreover, our analyses suggest that the key driver of both the periodic oscillations and chaotic fluctuations is the determinism of the cell cycle (Figures 6, S2F–S2H, S6E, and S6F).

We have focused here on the behavior of cells as they grow in lines in channels of the mother machine microfluidic device. The great advantage of this study system is that one can follow stress response dynamics of individual cells for relatively long periods in a bacterial population where cell-cell interactions are still preserved. These relatively long time series from single cells were important for our ability to both identify and describe chaos empirically. However, our work suggests that the chaotic behavior seen in this system also occurs in more complex and realistic growth conditions. When we study cells in 2D colonies, we also see unpredictable stress response dynamics, which are consistent with chaos (Figures 5D and 5E). It will be interesting to understand how chaotic processes are affected in even more complex growth environments, such as submerged biofilms. On the one hand, additional variation in the local density and arrangement of cells should increase the potential for chaos, while on the other the increasing capacity to scavenge H_2O_2 within larger communities may reduce stress levels and the duration of any chaotic dynamics.

Why have bacterial cells evolved to display such chaotic behavior? Our work shows that multiple processes interact to generate chaos, including the cell cycle and changes in local cell density. Another important factor is the strength of the stress response itself: a high expression rate and high catalytic efficiency of scavenging enzymes are critical to the transition from periodic oscillations to chaos (Figures 3 and 6). The evolution of chaotic behavior in the oxidative stress response may, therefore, lie in the benefits of a strong response for surviving stress, which is likely to provide a strong selective advantage to cells.[33,48] This importance of a strong response for chaos mirrors the classic theoretical results on chaos in population biology. There, models predict that high population growth rate is needed to generate chaotic dynamics[44] because this causes the population to overshoot equilibria and over-compensate.[49]

Chaos also results in a variable response among cells. An intriguing possibility, therefore, is that chaotic responses could have the benefit of diversifying cell behavior as a population bet-hedging strategy against unpredictable stresses.[50] Although microbes can harness intracellular molecular noise to generate phenotypic heterogeneity, deterministic chaos can achieve this

these peaks are absent for chaotic dynamics (teal) in the case of (A) growth rate perturbation (1,806 and 1,991 cells, respectively; n $\geq$ 3 repeats), (B) population size perturbation (1,003 and 1,440 cells, respectively; n $\geq$ 3 repeats), and (C) H_2O_2 concentration perturbation (1,003 and 1,361 cells, respectively; n $\geq$ 3 repeats).
(D) Bar plots show mean and standard deviation of maximal correlation dimension for GrxA traces of mother cells in experimental conditions shown in (A), (B), and (C) resulting in chaos (teal), or periodicity (black), as obtained from the Grassberger-Procaccia method.
(E) The periods of non-chaotic oscillations correlate with cell cycle duration (interdivision time) over a range of growth rates in simulations and experiments. Mean and standard deviation of interdivision time and the time of the first ACF peak for simulated GrxA dynamics (left, n = 3 simulations per condition) and experiments (right) with 100 μM H_2O_2 in M9 glycerol in 25-μm trenches (black), 50 μM H_2O_2 in M9 glucose in 10-μm trenches (dark blue), and 25 μM H_2O_2 in M9 glucose + 10% LB in 10-μm trenches (light blue) (966, 397, and 661 cells, respectively; n $\geq$ 3 repeats).
See also Figures S2 and S6 and Videos S4–S6.

without noise disturbing the response accuracy of each individual cell. In fact, our study showed that the conditions that lead to chaos are exactly those where bet-hedging is valuable in principle, namely at high stress levels and in cell populations but not in isolated cells.

Although our work is based upon the detailed study of one bacterial stress response, the existence of chaotic behavior may be widespread in cellular systems for related reasons.[3,51] Using a generalized model, we find that chaotic cell responses are possible whenever the absorbance of a stressor reduces a cell's growth rate and lowers the stressor concentration of the surrounding cells (Figure 3). The feedback between these effects creates spatio-temporal dynamics that amplify small perturbations, leading to chaotic behavior. Hence, the survival strategies of bacteria and other cells exposed to stressors—such as antibiotics, antimicrobial peptides, or reactive chemicals—all have the potential for deterministic chaos.

The study of chaos in biology has received considerable attention, and there are many potential examples where unpredictable dynamics have been observed that appear chaotic.[28] However, it remains challenging to identify chaos from either observational data or theoretical models alone. Here, we have presented a different strategy, which rests on the ability to manipulate chaos. In addition to the prediction of chaos and observational experiments, our model also correctly predicts the conditions where chaotic dynamics are lost. This close fit between model and measurements provides clear evidence for the existence of chaos in the single-cell dynamics of bacterial stress responses.

STAR★METHODS

Detailed methods are provided in the online version of this paper and include the following:

- KEY RESOURCES TABLE
- RESOURCE AVAILABILITY
  - Lead contact
  - Materials availability
  - Data and code availability
- EXPERIMENTAL MODEL AND SUBJECT DETAILS
  - Strains and plasmids
  - Media and growth conditions
- METHOD DETAILS
  - Microfluidics experiments
  - Microscopy
- QUANTIFICATION AND STATISTICAL ANALYSIS
  - Mother machine data processing and analysis
  - Microcolony image analysis
  - Chaos decision tree algorithm
  - Deterministic vs stochastic and live vs dead categorization of experimental traces
  - Autocorrelation analysis
  - Correlation Dimension to discriminate deterministic and stochastic processes
  - Lyapunov exponent computation
  - Quantitative model of the oxidative stress response in a bacterial population
  - Model simulations
  - Model of the oxidative stress response with noise

SUPPLEMENTAL INFORMATION

Supplemental information can be found online at https://doi.org/10.1016/j.cub.2023.11.002.

ACKNOWLEDGMENTS

We thank Philip Maini, Béla Novák, Nathalie Balaban, anonymous reviewers, and members of the Uphoff and Foster labs for their discussions and comments on the manuscript. Research in the Uphoff Lab is funded by a Wellcome Trust & Royal Society Sir Henry Dale Fellowship (206159/Z/17/Z), a Wellcome-Beit Prize (206159/Z/17/B), and a Research Prize Fellowship of the Lister Institute of Preventative Medicine. D.C. was supported by an Oxford-Indira Gandhi Scholarship funded by the Oxford India Center for Sustainable development. K.R.F. is supported by a European Research Council grant (787932) and by a Wellcome Trust investigator award (209397/Z/17/Z).

AUTHOR CONTRIBUTIONS

Conceptualization and design of study, D.C., K.R.F., and S.U.; data collection, D.C.; development of the computational model, D.C. and S.U.; data analysis and interpretation, D.C., K.R.F., and S.U.; writing of the article, D.C., K.R.F., and S.U.; supervision, K.R.F. and S.U.

DECLARATION OF INTERESTS

The authors declare no competing interests.

INCLUSION AND DIVERSITY

We support inclusive, diverse, and equitable conduct of research.

Received: June 14, 2023
Revised: September 29, 2023
Accepted: November 1, 2023
Published: November 28, 2023

REFERENCES

1. Strogatz, S.H. (2018). Nonlinear Dynamics and Chaos: With Applications to Physics, Biology, Chemistry, and Engineering (Westview Press).
2. Rogers, T.L., Johnson, B.J., and Munch, S.B. (2022). Chaos is not rare in natural ecosystems. Nat. Ecol. Evol. *6*, 1105–1111.
3. Karkaria, B.D., Manhart, A., Fedorec, A.J.H., and Barnes, C.P. (2022). Chaos in synthetic microbial communities. PLoS Comput. Biol. *18*, e1010548.
4. Allen, J.C., Schaffer, W.M., and Rosko, D. (1993). Chaos reduces species extinction by amplifying local population noise. Nature *364*, 229–232.
5. Cenci, S., and Saavedra, S. (2019). Non-parametric estimation of the structural stability of non-equilibrium community dynamics. Nat. Ecol. Evol. *3*, 912–918.
6. Doebeli, M., and Ispolatov, I. (2014). Chaos and unpredictability in evolution. Evolution *68*, 1365–1373.
7. Pearce, M.T., Agarwala, A., Agarwala, A., and Fisher, D.S. (2020). Stabilization of extensive fine-scale diversity by ecologically driven spatiotemporal chaos. Proc. Natl. Acad. Sci. USA *117*, 14572–14583.
8. Martinez de la Fuente, I., Martinez, L., Veguillas, J., and Aguirregabiria, J.M. (1996). Quasiperiodicity route to chaos in a biochemical system. Biophys. J. *71*, 2375–2379.
9. Zhdanov, V.P. (2011). Periodic perturbation of the bistable kinetics of gene expression. Phys. A *390*, 57–64.
10. Bi, P., Ruan, S., and Zhang, X. (2014). Periodic and chaotic oscillations in a tumor and immune system interaction model with three delays. Chaos *24*, 23101.
11. Heltberg, M., von Borries, M., Bendix, P.M., Oddershede, L.B., and Jensen, M.H. (2022). Temperature controls onset and period of NF-κB

CellPress
OPEN ACCESS

oscillations and can lead to chaotic dynamics. Front. Cell Dev. Biol. *10*, 910738.

12. Hirata, Y., and Aihara, K. (2012). Typical chaotic dynamics in squid giant axons. Procedia IUTAM *5*, 54–58.
13. Iasemidis, L.D., and Sackellares, J.C. (1996). Review: chaos theory and epilepsy. Neuroscientist *2*, 118–126.
14. Hirata, Y., Oku, M., and Aihara, K. (2012). Chaos in neurons and its application: perspective of chaos engineering. Chaos *22*, 47511.
15. Schweighofer, N., Doya, K., Fukai, H., Chiron, J.V., Furukawa, T., and Kawato, M. (2004). Chaos may enhance information transmission in the inferior olive. Proc. Natl. Acad. Sci. USA *101*, 4655–4660.
16. Heltberg, M.L., Krishna, S., Kadanoff, L.P., and Jensen, M.H. (2021). A tale of two rhythms: locked clocks and chaos in biology. Cell Syst. *12*, 291–303.
17. Gonze, D., Halloy, J., Leloup, J.C., and Goldbeter, A. (2003). Stochastic models for circadian rhythms: effect of molecular noise on periodic and chaotic behavior. C. R. Biol. *326*, 189–203.
18. Glass, L. (2009). Introduction to controversial topics in nonlinear science: is the normal heart rate chaotic? Chaos *19*, 28501.
19. Lloyd, A.L., and Lloyd, D. (1995). Chaos: its significance and detection in biology. Biol. Rhythm Res. *26*, 233–252.
20. Olsen, L.F., and Degn, H. (1977). Chaos in an enzyme reaction. Nature *267*, 177–178.
21. Kaity, B., Sarkar, R., Chakrabarti, B., and Mitra, M.K. (2018). Reprogramming, oscillations and transdifferentiation in epigenetic landscapes. Sci. Rep. *8*, 7358.
22. Zhang, Z., Ye, W., Qian, Y., Zheng, Z., Huang, X., and Hu, G. (2012). Chaotic motifs in gene regulatory networks. PLoS One *7*, e39355.
23. Glass, D.S., Jin, X., and Riedel-Kruse, I.H. (2021). Nonlinear delay differential equations and their application to modeling biological network motifs. Nat. Commun. *12*, 1788.
24. Chen, L., Hamarash, I.I., Jafari, S., Rajagopal, K., and Hussain, I. (2022). Various bifurcations in the development of stem cells. Eur. Phys. J. Spec. Top. *231*, 1015–1021.
25. Ahamed, T., Costa, A.C., and Stephens, G.J. (2021). Capturing the continuous complexity of behaviour in Caenorhabditis elegans. Nat. Phys. *17*, 275–283.
26. Becks, L., Hilker, F.M., Malchow, H., Jürgens, K., and Arndt, H. (2005). Experimental demonstration of chaos in a microbial food web. Nature *435*, 1226–1229.
27. Poon, C.S., and Barahona, M. (2001). Titration of chaos with added noise. Proc. Natl. Acad. Sci. USA *98*, 7107–7112.
28. Toker, D., Sommer, F.T., and D'esposito, M. (2020). A simple method for detecting chaos in nature. Commun. Biol. *3*, 11.
29. Storz, G., and Hengge, R. (2010). Bacterial Stress Responses (American Society for Microbiology Press).
30. Alnahhas, R.N., and Dunlop, M.J. (2023). Advances in linking single-cell bacterial stress response to population-level survival. Curr. Opin. Biotechnol. *79*, 102885.
31. Dawan, J., and Ahn, J. (2022). Bacterial stress responses as potential targets in overcoming antibiotic resistance. Microorganisms *10*, 1385.
32. Wakamoto, Y., Dhar, N., Chait, R., Schneider, K., Signorino-Gelo, F., Leibler, S., and McKinney, J.D. (2013). Dynamic persistence of antibiotic-stressed mycobacteria. Science *339*, 91–95.
33. Imlay, J.A. (2013). The molecular mechanisms and physiological consequences of oxidative stress: lessons from a model bacterium. Nat. Rev. Microbiol. *11*, 443–454.
34. Balázsi, G., Van Oudenaarden, A., and Collins, J.J. (2011). Cellular decision making and biological noise: from microbes to mammals. Cell *144*, 910–925.
35. Silander, O.K., Nikolic, N., Zaslaver, A., Bren, A., Kikoin, I., Alon, U., and Ackermann, M. (2012). A genome-wide analysis of promoter-mediated phenotypic noise in Escherichia coli. PLoS Genet. *8*, e1002443.
36. Patange, O., Schwall, C., Jones, M., Villava, C., Griffith, D.A., Phillips, A., and Locke, J.C.W. (2018). Escherichia coli can survive stress by noisy growth modulation. Nat. Commun. *9*, 5333.
37. Mitosch, K., Rieckh, G., and Bollenbach, T. (2017). Noisy response to antibiotic stress predicts subsequent single-cell survival in an acidic environment. Cell Syst. *4*, 393–403.e5.
38. De Martino, M., Ershov, D., van den Berg, P.J., Tans, S.J., and Meyer, A.S. (2016). Single-cell analysis of the Dps response to oxidative stress. J. Bacteriol. *198*, 1662–1674.
39. Mukhopadhyay, P., Zheng, M., Bedzyk, L.A., LaRossa, R.A., and Storz, G. (2004). Prominent roles of the NorR and Fur regulators in the Escherichia coli transcriptional response to reactive nitrogen species. Proc. Natl. Acad. Sci. USA *101*, 745–750.
40. Ma, M., and Eaton, J.W. (1992). Multicellular oxidant defense in unicellular organisms. Proc. Natl. Acad. Sci. USA *89*, 7924–7928.
41. Choudhary, D., Lagage, V., Foster, K.R., and Uphoff, S. (2023). Phenotypic heterogeneity in the bacterial oxidative stress response is driven by cell-cell interactions. Cell Rep. *42*, 112168.
42. Åslund, F., Zheng, M., Beckwith, J., and Storz, G. (1999). Regulation of the OxyR transcription factor by hydrogen peroxide and the cellular thiol-disulfide status. Proc. Natl. Acad. Sci. USA *96*, 6161–6165.
43. Wang, P., Robert, L., Pelletier, J., Dang, W.L., Taddei, F., Wright, A., and Jun, S. (2010). Robust growth of Escherichia coli. Curr. Biol. *20*, 1099–1103.
44. May, R.M. (1974). Biological populations with nonoverlapping generations: stable points, stable cycles, and chaos. Science *186*, 645–647.
45. Grassberger, P., and Procaccia, I. (1983). Characterization of strange attractors. Phys. Rev. Lett. *50*, 346–349.
46. Sandler, O., Mizrahi, S.P., Weiss, N., Agam, O., Simon, I., and Balaban, N.Q. (2015). Lineage correlations of single cell division time as a probe of cell-cycle dynamics. Nature *519*, 468–471.
47. Lagage, V., and Uphoff, S. (2020). Pulses and delays, anticipation and memory: seeing bacterial stress responses from a single-cell perspective. FEMS Microbiol. Rev. *44*, 565–571.
48. Lee, C., Lee, S.M., Mukhopadhyay, P., Kim, S.J., Lee, S.C., Ahn, W.S., Yu, M.H., Storz, G., and Ryu, S.E. (2004). Redox regulation of OxyR requires specific disulfide bond formation involving a rapid kinetic reaction Path. Nat. Struct. Mol. Biol. *11*, 1179–1185.
49. Deangelis, D.L., and Waterhouse, J.C. (1987). Equilibrium and nonequilibrium concepts in ecological models. Ecol. Monogr. *57*, 1–21.
50. Ackermann, M. (2015). A functional perspective on phenotypic heterogeneity in microorganisms. Nat. Rev. Microbiol. *13*, 497–508.
51. Heltberg, M.L., Krishna, S., and Jensen, M.H. (2019). On chaotic dynamics in transcription factors and the associated effects in differential gene regulation. Nat. Commun. *10*, 71.
52. Schindelin, J., Arganda-Carreras, I., Frise, E., Kaynig, V., Longair, M., Pietzsch, T., Preibisch, S., Rueden, C., Saalfeld, S., Schmid, B., et al. (2012). Fiji: an open-source platform for biological-image analysis. Nat. Methods *9*, 676–682.
53. Ollion, J., Elez, M., and Robert, L. (2019). High-throughput detection and tracking of cells and intracellular spots in mother machine experiments. Nat. Protoc. *14*, 3144–3161.
54. Zaslaver, A., Bren, A., Ronen, M., Itzkovitz, S., Kikoin, I., Shavit, S., Liebermeister, W., Surette, M.G., and Alon, U. (2006). A comprehensive library of fluorescent transcriptional reporters for Escherichia coli. Nat. Methods *3*, 623–628.
55. Balleza, E., Kim, J.M., and Cluzel, P. (2018). Systematic characterization of maturation time of fluorescent proteins in living cells. Nat. Methods *15*, 47–51.
56. Lagage, V., Chen, V., and Uphoff, S. (2023). Adaptation delay causes a burst of mutations in bacteria responding to oxidative stress. EMBO Rep. *24*, e55640.

57. Sliusarenko, O., Heinritz, J., Emonet, T., and Jacobs-Wagner, C. (2011). High-throughput, subpixel precision analysis of bacterial morphogenesis and intracellular spatio-temporal dynamics. Mol. Microbiol. *80*, 612–627.

58. Susman, L., Kohram, M., Vashistha, H., Nechleba, J.T., Salman, H., and Brenner, N. (2018). Individuality and slow dynamics in bacterial growth homeostasis. Proc. Natl. Acad. Sci. USA *115*, E5679–E5687.

59. Yang, D., Jennings, A.D., Borrego, E., Retterer, S.T., and Männik, J. (2018). Analysis of factors limiting bacterial growth in PDMS mother machine devices. Front. Microbiol. *9*, 871.

60. Seaver, L.C., and Imlay, J.A. (2001). Hydrogen peroxide fluxes and compartmentalization inside growing Escherichia coli. J. Bacteriol. *183*, 7182–7189.

61. Uhl, L., and Dukan, S. (2016). Hydrogen peroxide induced cell death: the major defences relative roles and consequences in E. coli. PLoS One *11*, e0159706.

62. Pillay, C.S., Eagling, B.D., Driscoll, S.R.E., and Rohwer, J.M. (2016). Quantitative measures for redox signaling. Free Radic. Biol. Med. *96*, 290–303.

63. Murray, J.D. (2002). Mathematical biology I. An introduction. Interdiscip. Appl. Math. *17*, 551.

64. Bower, J.M., and Bolouri, H. (2001). Computational Modeling of Genetic and Biochemical Networks (MIT Press).

65. Angelova, M., and Ben-Halim, A. (2011). Dynamic model of gene regulation for the lac operon. J. Phys. Conf. Ser. *286*, 12007.

66. Stark, H., and Woods, J.W. (2002). Probability and Random Processes with Applications to Signal Processing (Prentice Hall), p. 706.

67. Kloeden, P.E., and Platen, E. (1992). Numerical Solution of Stochastic Differential Equations (Springer).

68. Shaikhet, L. (2011). Lyapunov Functionals and Stability of Stochastic Difference Equations (Springer Science & Business Media).

STAR★METHODS

KEY RESOURCES TABLE

REAGENT or RESOURCE	SOURCE	IDENTIFIER
**Bacterial Strains**		
AB1157, Δ*flhD*, P_{RNAI}-mKate2, mutL-mYPet (SU178)	Choudhary et al.[41]	N/A
AB1157, Δ*flhD*, P_{RNAI}-mKate2, mutL-mYPet, carrying pUA139 P*grxA*-SCFP3A Kan (SU777)	Choudhary et al.[41]	N/A
**Chemicals, Peptides, and Recombinant Proteins**		
M9 minimal salts 5x	Sigma	Product Number: M9956
MEM amino acids	Gibco	Catalog number: 11130-036
L-Proline	Biochemica	Reference Number: A3453,0100
Thiamine	Biochemica	Reference Number: A0955,0050
Pluronic F-127	Sigma	Product Number: P2443-250G
Propidium iodide	Sigma	Product Number: P4170
30% W/W solution of H_2O_2	Sigma	Product Number: H1009-100mL
Kanamycin	Sigma	Product Number: A1493
Agarose	Bio-Rad	Product Number: 1613100
PDMS	Univar Specialty Consumables Ltd	Dowsil / Dow Corning Sylgard 184 Kit 1.1kg
**Software and Algorithms**		
MATLAB	Mathworks	Mathworks.com
BACMMAN	Fiji,[52] Ollion et al.[53]	github.com/jeanollion/bacmman
Python	Spyder	anaconda.com
Python code for model simulations and experimental data analysis	This study	github.com/divyachoudhary2809/Chaos
**Deposited Data**		
Raw data collected	This study	Oxford Research Archive: https://doi.org/10.5287/ora-b7dw9pmqd

RESOURCE AVAILABILITY

Lead contact

Further information and requests for resources and reagents should be directed to and will be fulfilled by the lead contact, Stephan Uphoff (stephan.uphoff@bioch.ox.ac.uk).

Materials availability

The study did not generate new unique reagents.

Data and code availability

All data reported in this paper will be shared by the lead contact upon request.

All the raw data collected for analysis in this study is freely and openly available on the Oxford Research Archive: https://doi.org/10.5287/ora-b7dw9pmqd. Custom-built python codes for model simulations and experimental data analysis are available on Github https://github.com/divyachoudhary2809/Chaos.

Any further information about data and code is available upon request by the lead contact.

EXPERIMENTAL MODEL AND SUBJECT DETAILS

We performed experiments with bacterial strains that were derived from *E. coli* K-12 AB1157. The description of genetic modification and growth conditions is described in sections below.

Strains and plasmids

All experiments were performed with a strain derived from *E. coli* K12 AB1157 that was previously described in Choudhary et al.[41] The strain constitutively expressed P_{RNAI}-mKate2 fluorescent marker for cell segmentation analysis and the *flhD* gene was deleted to inhibit flagellar motility allowing growth in mothermachine microfluidic chips. The OxyR response reporter plasmid carrying P*grxA*-SCFP3 was derived from an *E. coli* promoter library of pSC101 plasmids.[54] Each plasmid in the library contains the promoter region of a specific gene or operon in front of GFPmut2 fluorescent protein. We changed the GFPmut2 to the fast-maturing cyan fluorescent protein SCFP3[55] using Gibson Assembly (NEB). The promoter region was confirmed by sequencing and the plasmid was transformed yielding strain SU777 (AB1157, Δ*flhD*, P_{RNAI}-mKate2, mutL-mYPet, carrying plasmid pUA139 P*grxA*-SCFP3 with kanamycin resistance gene). Presence of the expected fluorescent protein signal was verified by taking microscopy snapshots.

Media and growth conditions

Cells were grown at 37°C for all experiments. Cells were streaked from glycerol stocks stored at -80°C on LB agarose plates with 25 μg/mL kanamycin. A single colony was picked and grown overnight shaking in 4 mL M9 minimal media. This media was prepared with M9 salts (15 g/L KH_2PO_4, 64 g/L Na_2HPO_4, 2.5 g/L NaCl, and 5.0 g/L NH_4Cl), 2 mM $MgSO_4$, 0.1 mM $CaCl_2$, 0.5 mg/mL thiamine, MEM amino acids, 0.1 mg/mL L-proline, and 0.2% carbon source (glucose or glycerol). The next day, overnight culture was diluted 1:50 and grown shaking to OD600 ~0.3 in 4 mL M9 minimal media. For loading cells in microfluidic chips, 0.85 mg/mL Pluronic F127 was added to the media to avoid cell aggregation. For experiments done under hydrogen peroxide treatment, the specific concentration of H_2O_2 was added to the growth media immediately before the start of the experiment. LB (10% V/V) was added to M9 glucose media for certain experiments, as specified in the figures.

METHOD DETAILS

Microfluidics experiments

Mother machine chip preparation

Single-cell imaging was performed using the 'mother machine' microfluidic device as described in [43,56]. The chip has a main channel for flow of media, branching into perpendicular growth channels (here called 'growth trenches') of dimension 1.2 μm width and 1.2 μm height and 25 μm length. The chips were made of polydimethysiloxane (PDMS, Dow Corning Sylgard 184 kit) polymer using a silicon wafer mold (Conscience). A 1:10 solution of polymerising agent and PDMS monomer were rigorously mixed and then poured onto the silicon wafer. This was placed in a vacuum chamber and pressurised to remove air bubbles. The device was then heated at 65°C in an oven for 2 hours to polymerise. For each experiment, one chip was cut out using a scalpel, and holes for inlet and outlet were inserted using a 0.75 mm biopsy puncher. The device was cleaned using 100% ethanol and dried with nitrogen gas. The cleaning was repeated 3 times. The PDMS chip was bonded on a glass coverslip (thickness No 1.5). These coverslips were first cleaned by sonication with acetone for 20 mins followed by isopropanol for 20 min, and then dried with nitrogen gas. The cleaned coverslip and PDMS chip were exposed to air plasma for 2 min and bonded at 95°C for 30 min.

Where indicated, a different silicon wafer was used to generate mother machine chips with shorter trenches of 10 μm length and 1.2 μm width and 1.2 μm height. Here a "negative" mould was prepared first using PDMS as intermediate from a silicon wafer (by mixing monomer and curing agent 1:5) that was then used to prepare the "positive" chips using the method explained above.

Mother machine setup

1 mL of exponentially growing cells were spun down for 2 min at 6000 rpm. Cells in the pellet were resuspended in 100 μL of the supernatant and loaded in the microfluidic chips by pipetting through the inlet. The chip was then inserted into a custom-built centrifuge holder and spun at 5000 rpm for 10 min to aid the loading of cells into the growth trenches. 50 mL syringes were filled with M9 minimal media containing Pluronic F127 and H_2O_2 as indicated. The syringes were attached to silicon tubing (Tygon) and loaded onto syringe pumps (NewEra SyringePumpPro) to deliver media into chips at a constant flow rate of 2.5 mL per hour. Cells were initially grown without H_2O_2 for ~3 hours before switching the inlet media to a syringe containing H_2O_2.

Microcolony chip preparation

E. coli microcolonies were grown on 1% agarose pads made with M9 glucose + 10% LB media. The procedure of preparing these pads is shown in Figure S5A. Melted agarose solution was poured into the top of an empty 50 mL syringe plunger head wrapped with adhesive tape to act like a container. A glass cover slip was then placed on top of the taped cylinder. After the agarose was set, the cover slip was removed and 1 μl spots of overnight culture were dropped on the flat agarose surface and grown for 2 hours at 37°C. After 2 hours, the agarose was removed from the plunger head, thus leaving a conical dip in the agarose that acted as reservoir for adding H_2O_2 treatment solution. The agarose pad was inverted to sandwich the cells between the agarose and a cover slip for imaging. A needle was inserted through the adhesive tape to continuously flow in growth medium with H_2O_2 using syringe pumps. The medium dripped onto the conical dip and diffused through the agarose to reach the cells below.

Microscopy

Time-lapse microscopy of cells in mother machine chips

Time-lapse imaging was performed using a Nikon Ti-E inverted fluorescence microscope equipped with 100x NA 1.40 immersion oil objective, motorized stage, sCMOS camera (Hamamatsu Flash 4), LED excitation source (Lumencor SpectraX), and operated with a perfect focus system. Exposure times were 100 ms for P_{RNAI}-mKate2 (λ = 555 nm) and 75 ms for sCFP3 reporter (λ = 440 nm) using

50% of maximal LED excitation intensities. The excitation and emission lights were separated using a triband dichroic and individual emission filters. The microscope chamber (Okolabs) was maintained at 37°C throughout the experiments. Images were captured every 3 min for the 2 emission channels.

Time-lapse microscopy of cells in microcolonies

Time lapse imaging was performed on a Nikon Ti-E microscope equipped with a 100x NA 1.45 oil immersion objective, motorised stage, sCMOS camera (Photometrics Prime95B), LED excitation source (Lumencor SpectraX) and perfect focus system. Exposure times were 100 ms for P_{RNAI}-mKate2 (λ = 555 nm) and 75 ms for sCFP3 reporter (λ = 440 nm) using 50% of maximal LED excitation intensities. The microscope chamber (Okolabs) was maintained at 37°C throughout the experiments. Images were captured every 3 minutes with phase contrast and the two fluorescence channels.

QUANTIFICATION AND STATISTICAL ANALYSIS

Mother machine data processing and analysis

Time-lapse microscopy data were saved as .nd2 files and visualized in Fiji.[52] The data were processed using the BACMMAN plugin in Fiji as described in [53] and further analysed using custom Python and MATLAB scripts. Images were first pre-processed by BACMMAN using the P_{RNAI}-mKate2 fluorescence channel to stack all individual growth trenches and correct for experimental drift in x-y coordinates and image rotation. The outlines of cells in the growth trenches were then jointly segmented and tracked over time based on the P_{RNAI}-mKate2 fluorescence signal. The traces were visually inspected and manually corrected for errors in segmentation or lineage tracing using the BACMMAN software. The SCFP3 fluorescence was extracted by overlaying the cell masks from the P_{RNAI}-mKate2 channel onto the SCFP3 channel and computing the mean intensity over the cell area. BACMMAN generated output in 3 excel files containing cell growth characteristic, P_{RNAI}-mKate2 intensity data and SCFP3 intensity data. These files were then further analyzed using Python and MATLAB code as described below.

Microcolony image analysis

Segmentation of cells growing in microcolonies was performed based on the P_{RNAI}-mKate2 fluorescence signal and using the MicrobeTracker tool in MATLAB[57] followed by manual correction of the segmentation masks. These outlines were then applied to the CFP channel and a MATLAB script was used to quantify the average intensity per cell area. Cell lineage tracing was performed manually and custom python code was used to plot CFP intensity traces.

Chaos decision tree algorithm

To categorise if the fluctuations in P*grxA*-SCFP3 traces of individual mother cells are deterministic or stochastic, we applied the 'Chaos decision tree algorithm' as described by Toker et al.[28] The pipeline is available as MATLAB code. Briefly, the algorithm tests for stochasticity by computing the permutation entropy using the cyclic phase permutation algorithm. The permutation entropy quantifies the extent to which the values in a trace are ordered or random in time. The value of the permutation entropy for the original trace is compared to many randomly shuffled versions of the same trace in which the temporal order of the data points is removed while the mean and standard deviation are maintained. If the fluctuations are stochastic, then the permutation entropy is similar for the original and shuffled traces; otherwise the fluctuations are classified as deterministic. The chaos decision tree further tests for stationarity. i.e. whether statistical properties like the mean and standard deviation of a trace do not change over time; else the trace is classified as non-stationary.

Deterministic vs stochastic and live vs dead categorization of experimental traces

The MATLAB code described in Toker et al.[28] as explained in the section above was used to categorise P*grxA*-SCFP3 traces of individual mother cells as showing deterministic or stochastic fluctuations. Traces were analysed at steady-state from 1-hour after the start of H_2O_2 treatment. The traces were pre-processed with a moving-mean filter using a window of 3 time points (i.e. 9 min) twice. The rationale for this is that we are principally interested in the large-scale response fluctuations on the time-scale of the cell cycle (~50 to 100 min, depending on conditions). Only traces with at least 5.5 hours of data were included in this analysis (length of data required for entropy calculation in the pipeline).

P*grxA*-SCFP3 traces were further categorised as originating from live or dead mother cells. A cell was considered dead if the length growth rate as computed by BACMMAN was below 0.0024 min^{-1}.

Autocorrelation analysis

Autocorrelation analysis was performed to distinguish between periodic and chaotic fluctuations of the P*grxA*-SCFP3 traces of individual mother cells. Traces with at least 2-hours of data were analysed at steady-state from 1-hour after the start of H_2O_2 treatment. The traces were pre-processed with a moving-mean filter using a window of 3 frames (i.e. 9 min) twice. Due to the slower growth of cells in M9 glycerol media, a window of 6 frames was used and traces were analysed at steady-state from 2-hours after the start of H_2O_2 treatment. ACF curves were computed from the DPgrxA difference signal, which was calculated by subtracting the P*grxA*-SCFP3 values of consecutive frames. The Python stattools.acf function from the statsmodel library was used to output the autocorrelation value over a range of lag times. Mean ACF curves were computed by averaging the ACF values from the single-cell traces at each lag time. A peak finding algorithm was applied to the mean ACF curves to quantify the period of non-chaotic P*grxA*-SCFP3

traces. This was done in python using the find_peaks function in the scipy.signal library with a prominence of 0.2. ACF curves for simulated GrxA traces were computed during steady-state from 700 to 5200 min after start of H_2O_2 treatment at t = 50 min. The analysis was done as for experimental data but without moving-mean filtering.

Correlation Dimension to discriminate deterministic and stochastic processes

We used the Grassberger - Procaccia algorithm[45] to estimate the correlation dimension (D_{corr}) of the GrxA response dynamics, which can be understood as the effective number of dynamic variables that generate the response fluctuations. A large number implies a stochastic process whereas a low number results from a deterministic process.[46]

To compute the correlation dimension, we followed the procedure described in Sandler et al.[46] For a given trace of {$GrxA_n$} where n is any time point, the algorithm constructs a vector {$GrxA_n$, $GrxA_{n+1}$, $GrxA_{n+2}$,..., $GrxA_{n+E-2}$, $GrxA_{n+E-1}$} considering them in E-dimensional space (E is also called embedding dimension). For a data set with N data points in an embedding dimension E, the correlation sum $C(r)$ is quantified to then compute d_{corr}:

$$C(E,r) = \frac{2}{N(N-1)} \sum_{i=1}^{N} \sum_{j=i+1}^{N} \Theta(r - |x_i - x_j|)$$

The Heaviside step function $\Theta(x)$ ($\Theta(x) = 1$ if $x > 0$ and $\Theta(x) = 0$ if $x \leq 0$) is used to compute the fraction of data points x_i and x_j that are within a distance r of each other. The values of r were evenly spaced in log scale from 0.01σ to 3σ, where σ is the standard deviation of the fluctuation of the given GrxA trace.

The correlation dimension $C(r)$ follows a power law for small r, such that $C(r)\alpha r^{D_{corr}}$. Therefore, D_{corr} can be estimated from the slope of a log-log plot of $C(r)$ versus r. $C(r)$ grows monotonically with r.

The D_{corr} obtained for different values of E is plotted against E. For a deterministic process, the D_{corr} vs E plot saturates and the saturating value of D_{corr} corresponds to the effective dimension of the dynamic process, i.e. the number of dynamic variables that determine the response dynamics. In contrast, a truly stochastic process yields a straight line for D_{corr} vs E, with $E = D_{corr}$ for any value of dimension E, implying that the process is determined by an infinite number of dynamic variables.

We applied a moving-average filter with a filtering window of 3 time points (i.e. 9 minutes) to smooth experimental P*grxA*-SCFP3 traces before applying the Grassberger – Procaccia algorithm. The rationale for this is that we are principally interested in the large-scale response fluctuations on the time-scale of the cell cycle (~50 to 100 min, depending on conditions). Nevertheless, applying the Grassberger – Procaccia algorithm to experimental data without smoothing also produced a low correlation dimension (Figures S4H and S4I), consistent with the dynamics being overall deterministic.

Lyapunov exponent computation

Lyapunov exponent was computed for simulated GrxA traces of mother cells (positioned at the closed end of growth trenches) during steady-state from 700 to 5200 min after start of H_2O_2 treatment. The traces were normalized in MATLAB using the "normalize" function that rescales the data with mean of 0 and standard deviation of 1. The Lyapunov exponent was computed in MATLAB using the "phaseSpaceReconstruction" function and "lyapunovExponent" function with fs = 10.

Quantitative model of the oxidative stress response in a bacterial population

Cell growth model

The growth of rod-shaped *E. coli* cells was described by an adder model with equal length added to the cell over time between successive division events.[58] The elongation rate g determines the increase in cell length l from time t to $t+1$:

$$l_{t+1} = l_t \cdot (1+g) \quad \text{(Equation 1)}$$

The cell divides into 2 daughter cells of equal length when the total cell length added from the birth length exceeds 2 μm. We modelled the inhibition of the cell elongation rate by H_2O_2 as a sigmoidal decay:

$$g\left([H_2O_2]_{cell}\right) = g_0 \cdot \left(1 - \frac{1}{1+10^{-c_1\left([H_2O_2]_{cell} - c_2\right)}}\right) \quad \text{(Equation 2)}$$

where g_0 is the elongation rate without treatment, $[H_2O_2]_{cell}$ is the intracellular H_2O_2 concentration, and the sigmoidal decay factors c_1, c_2 determine the sensitivity of the elongation rate to $[H_2O_2]_{cell}$.

Cell-cell interaction model

Scavenging of H_2O_2 results in concentration gradients that are determined by the spatial arrangement of cells and the diffusion of H_2O_2 with diffusion coefficient D. Hence, $[H_2O_2]_{external}$ is a function of a cell's position x along the length of the growth trench. The spatial arrangement was modelled as a one-dimensional colony of rod-shaped cells with radius R_C in cuboid growth trenches with square cross section W^2. This is analogous to the growth trenches in a mother machine device[41] with H_2O_2 treatment entering through the open end of the trench (where $x = 0$). The scavenging rate of H_2O_2 is limited by the absorption of H_2O_2 across the cell envelope[41] with absorption rate constant k_{abs}. We modelled the spatial profile of $[H_2O_2]_{external}$ based on the reaction-diffusion equation, using a similar approach as described in Yang et al.[59]:

$$\frac{\partial[H_2O_2]_{external}}{\partial t} = \left(W^2 - \pi R_c^2\right) D \frac{\partial^2 [H_2O_2]_{external}}{\partial x^2} - 2\pi R_c k_{abs} [H_2O_2]_{external} \qquad \text{(Equation 3)}$$

We first consider the profile of $[H_2O_2]_{external}$ along the length of a single cylinder-shaped cell of length l_t (the hemispherical caps at the cell poles will be considered below). At each position x, $(W^2 - \pi R_c^2)$corresponds to the area around the cell where H_2O_2 diffuses through the trench. The circular perimeter of the cell where H_2O_2 is absorbed is $2\pi R_c$. At steady-state, $\frac{\partial[H_2O_2]_{external}}{\partial t} = 0$. Therefore, the reaction-diffusion equation becomes:

$$\left(W^2 - \pi R_c^2\right) D \frac{\partial^2 [H_2O_2]_{external}}{\partial x^2} = 2\pi R_c k_{abs} [H_2O_2]_{external} \qquad \text{(Equation 4)}$$

To solve this equation for $[H_2O_2]_{external}$, we introduce the coefficient λ:

$$\frac{\partial^2 [H_2O_2]_{external}}{\partial x^2} = \frac{1}{\lambda^2} [H_2O_2]_{external} \qquad \text{(Equation 5)}$$

with $\lambda = \sqrt{\frac{(W^2 - \pi R_c^2)\, D}{2\pi R_c k_{abs}}}$

We assume the non-adsorbing boundary condition at x =l_t.

The solution to this equation is:

$$[H_2O_2]_{external}(x) = [H_2O_2]_{external,0} \frac{\cosh\left(\frac{x - l_t}{\lambda}\right)}{\cosh\left(\frac{l_t}{\lambda}\right)} \qquad \text{(Equation 6)}$$

Here, $[H_2O_2]_{external,0}$ is the concentration at $x = 0$.

We modify Equation 7 to account for the shape of *E. coli* cells as cylinders capped with hemispheres. First, we multiply Equation 5 by the cell length l_t:

$$\left(W^2 - \pi R_c^2\right) \cdot l_t \cdot D \cdot \frac{\partial^2 [H_2O_2]_{external}}{\partial x^2} = 2\pi R_c \cdot k_{abs} \cdot l_t \cdot [H_2O_2]_{external} \qquad \text{(Equation 7)}$$

Compared to the cylinder geometry, the free volume in the trench through which H_2O_2 diffuses increases by $2\pi R_c^3 - \frac{4}{3}\pi R_c^3$ due to the two hemispherical caps. The surface area for H_2O_2 absorption is unchanged. Hence:

$$\left(W^2 l_t - \pi R_c^2 l_t + \left(2\pi R_c^3 - \frac{4}{3}\pi R_c^3\right)\right) \cdot D \cdot \frac{\partial^2 [H_2O_2]_{external}}{\partial x^2} = 2\pi R_c \cdot k_{abs} \cdot l_t \cdot [H_2O_2]_{external} \qquad \text{(Equation 8)}$$

The solution to the equation is $[H_2O_2]_{external} = [H_2O_2]_{external,0} \frac{\cosh\left(\frac{x - l_t}{\lambda'}\right)}{\cosh\left(\frac{l_t}{\lambda'}\right)}$ (Equation 9)

Where, $\lambda' = \sqrt{\frac{\left(W^2 l_t - \pi R_c^2 l_t + \left(\frac{2}{3}\pi R_c^3\right)\right) D}{2\pi R_c k_{abs} l_t}}$

Oxidative stress response model

The oxidative stress response was modelled based on mass action kinetics using a set of 5 coupled ordinary differential equations (ODEs) to predict the dynamics of gene expression and intracellular H_2O_2 concentration ($[H_2O_2]_{cell}$) for a given external H_2O_2 concentration ($[H_2O_2]_{external}$). This is illustrated in the following schematic:

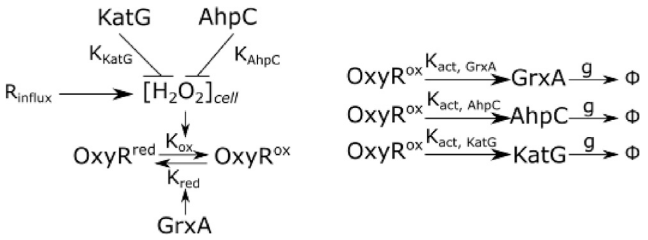

$[H_2O_2]_{cell}$ oxidises the transcription factor OxyR from its reduced to oxidised form, where K_{ox} is the 2nd-order oxidation rate constant. The total OxyR concentration was assumed constant, such that $[OxyR]_{Ox} = [OxyR]_{total} - [OxyR]_{Red}$. Oxidised OxyR is reduced by GrxA with Michaelis-Menten kinetics where K_{red} is the catalytic rate constant and h_{OxyR} is the Michaelis constant. Hence:

$$\frac{d[OxyR]_{Red}}{dt} = -K_{ox} \cdot [OxyR_{Red}] \cdot [H_2O_2]_{cell} + K_{red} \cdot [GrxA] \cdot \left(\frac{[OxyR]_{total} - [OxyR]_{Red}}{([OxyR]_{total} - [OxyR]_{Red}) + h_{OxyR}}\right) \qquad \text{(Equation 10)}$$

The OxyR regulon includes numerous genes involved in various aspects of oxidative stress tolerance. We reduced this system to the two key H_2O_2 scavenging enzyme genes *katG* and *ahpC*, and the glutaredoxin-1 *grxA*. Each gene has a basal expression rate ($R_{grxA,basal}$, $R_{katG,basal}$, $R_{ahpC,basal}$) and an inducible expression rate that depends on the concentration of oxidised OxyR ($[OxyR]_{total} - [OxyR]_{Red}$). The maximal induced-expression rates are given by $K_{grxA,act}$, $K_{katG,act}$, $K_{ahpC,act}$. The parameters $h_{grxA,act}$, $h_{katG,act}$, $h_{ahpC,act}$ define the concentrations of $[OxyR]_{Ox}$ that give half-maximal induction of each gene. Gene expression is counteracted by dilution due to cell growth with rate g, given by the growth model (Equation 2). The gene expression dynamics follow:

$$\frac{d[GrxA]}{dt} = R_{grxA,basal} + K_{grxA,act} \cdot \left(\frac{[OxyR]_{total} - [OxyR]_{Red}}{([OxyR]_{total} - [OxyR]_{Red}) + h_{grxA,act}} \right) - g \cdot [GrxA] \tag{Equation 11}$$

$$\frac{d[KatG]}{dt} = R_{katG,basal} + K_{katG,act} \cdot \left(\frac{[OxyR]_{total} - [OxyR]_{Red}}{([OxyR]_{total} - [OxyR]_{Red}) + h_{katG,act}} \right) - g \cdot [KatG] \tag{Equation 12}$$

$$\frac{d[AhpC]}{dt} = R_{aphC,basal} + K_{ahpC,act} \cdot \left(\frac{[OxyR]_{total} - [OxyR]_{Red}}{([OxyR]_{total} - [OxyR]_{Red}) + h_{ahpC,act}} \right) - g \cdot [AhpC] \tag{Equation 13}$$

The intracellular H_2O_2 concentration is determined by the influx of external H_2O_2 with rate $R_{influx} \cdot [H_2O_2]_{external}$, a basal endogenous production rate $R_{H_2O_2,basal}$, and scavenging by catalase and peroxidase enzymes with Michaelis-Menten kinetics where K_{AhpC}, K_{KatG} are the catalytic rate constants and h_{AhpC}, h_{KatG} are the Michaelis constants:

$$\frac{d[H_2O_2]_{cell}}{dt} = R_{influx} \cdot [H_2O_2]_{external} + R_{H_2O_2,basal} - K_{Ahpc} \cdot [\mathrm{AhpC}] \cdot \left(\frac{[H_2O_2]_{cell}}{[H_2O_2]_{cell} + h_{AhpC}} \right) - K_{KatG} \cdot [\mathrm{KatG}] \cdot \left(\frac{[H_2O_2]_{cell}}{[H_2O_2]_{cell} + h_{KatG}} \right) \tag{Equation 14}$$

General stress response model

We formulated a general model to study the emergence of chaos in the response to a generic toxin. The purpose of this model was to abstract as many of the molecular details as possible and explore if a simple gene regulatory circuit is still capable of producing chaotic dynamics when coupled with the growth model and the cell-cell interaction model described above. The intracellular toxin concentration $[Toxin]_{cell}$ is determined by the influx of external toxin with rate constant R_{influx}, and detoxification by an enzyme E where K_{cat} is the catalytic rate constant and h_E is the Michaelis constant:

$$\frac{d[Toxin]_{cell}}{dt} = R_{influx}[Toxin]_{external} - K_{cat}[\mathrm{E}] \left(\frac{[Toxin]_{cell}}{[Toxin]_{cell} + h_E} \right) \tag{Equation 15}$$

The detoxifying enzyme is produced at an inducible expression rate that depends on the intracellular toxin concentration. The maximal induced-expression rate is K_{act} and h_{act} defines the toxin concentration that gives half-maximal gene induction. Enzyme expression is counteracted by dilution due to cell growth with rate g, given by the growth model (Equation 2).

$$\frac{d[E]}{dt} = K_{act} \left(\frac{[Toxin]_{cell}}{[Toxin]_{cell} + h_{act}} \right) - g[E] \tag{Equation 16}$$

As above, the cell elongation rate depends on the intracellular toxin concentration:

$$g\left([Toxin]_{cell} \right) = g_0 \cdot \left(1 - \frac{1}{1 + 10^{c_1\left([Toxin]_{cell} - c_2\right)}}\right) \tag{Equation 17}$$

Model simulations

Software

Simulations of the model were performed using custom-written Python code. The following libraries were used: pandas, numpy, math, scipy, random, and matplotlib.

Growth model parametrisation

Under the experimental conditions of our study, the mean elongation rates of *E. coli* cells growing without H_2O_2 in M9 glycerol, M9 glucose and M9 glucose + 10% LB media are $g_0 = 0.025\,\mathrm{min}^{-1}, 0.042\,\mathrm{min}^{-1}$ and $0.065\,\mathrm{min}^{-1}$ respectively. For the sigmoidal function describing the sensitivity of elongation rate to H_2O_2, the growth rate reduces to half when $[H_2O_2]_{cell} = c_2 = 10^{-4}$ µM, with growth stalling at $[H_2O_2]_{cell} \sim 2c_2$. The value c_2 was chosen in the sub-nanomolar range as *E. coli* cells can tolerate up to nanomolar $[H_2O_2]_{cell}$ in the absence of exogenous H_2O_2.[60] The steepness of the sigmoidal decay is given by parameter c_1 and was refined to match the variation of elongation rates for cells at different positions in a growth trench under H_2O_2 treatment observed in the mother machine experiments. To this end, we varied c_1 over five order of magnitude from $200\,\mu\mathrm{M}^{-1}$ and minimized the value of mean absolute error

(MAE) between g_{model} and $g_{experimental}$. For a given value of c_1, the MAE was estimated for steady-state elongation rate values obtained for cells at different positions in trenches growing in M9 glucose and treated with various concentrations of H_2O_2 (12.5 μM, 25 μM, 37.5 μM, 50 μM, 62.5 μM, 75 μM, 82.5 μM, 100 μM and 500 μM).

$$MAE = \left|\frac{g_{model,i,cell\_position} - g_{experimental,i,cell\_position}}{g_{experimental,i,cell\_position}}\right|$$

Where i denotes the different H_2O_2 concentrations and *cell_position* denotes the position of cells in the trench from the open end. The MAE plotted against different values of c_1 showed a minimum at $2 \cdot 10^4 \mu M^{-1}$ [Figures S3C and S3D].

Cell-cell interaction model parametrisation

The reaction-diffusion Equation 9 was solved with a cell radius of R_c= 0.575 μm and growth trench width W = 1.2 μm. The diffusion coefficient of H_2O_2 in water is $D = 10^{-9} m^2/s$. The H_2O_2 absorption rate[60] is $k_{abs} = 1.6 \cdot 10^{-5} m/s$.

Oxidative stress response model parametrisation

The model was parametrised using the following literature values:

$K_{Ahpc} = 660 s^{-1}$ (Ref [61]), $h_{AhpC} = 1.2\ \mu M$ (Ref [61]), $K_{KatG} = 490000 s^{-1}$ (Ref [61]), $h_{KatG} = 5900\ \mu M$ (Ref [61]), $R_{H_2O_2,basal} = 0.02\ \mu M\ min^{-1}$ (Ref [60,61]), $K_{ox} = 0.1 \mu M^{-1} s^{-1}$ (Ref [62]), $K_{red} = 8 \mu M s^{-1}$ (Ref [62]), $h_{OxyR} = 2583\ \mu M$ (Ref [62]), $[OxyR]_{total}$=1 μM (Ref [62]).

The gene regulatory parameters were matched to the experimental data presented in this paper and in Choudhary et al.[41]:

$$h_{AhpC,act} = 0.1\ \mu M, h_{KatG,act} = 0.18\ \mu M, h_{GrxA,act} = 0.1\ \mu M, K_{AhpC,act} = 0.2\ \mu M\ min^{-1},$$

$$K_{KatG,act} = 0.15\ \mu M\ min^{-1}, K_{GrxA,act} = 0.1\ \mu M\ min^{-1}, R_{grxA,basal} = 0\ \mu M\ min^{-1}, R_{katG,basal} = 0\ \mu M\ min^{-1}, R_{aphC,basal} = 0.01\ \mu M\ min^{-1}, R_{influx} = 1 min^{-1}$$

We estimated the calibration factor $k_{GrxA,calibration}$ which allowed to compare the P*grxA-SCFP3* intensity from experiments to the GrxA concentrations in the model simulations, such that:

$$GrxA_{model,calibrated} = k_{GrxA,calibration} \cdot GrxA_{model} = GrxA_{experimental}$$

We varied $k_{GrxA,calibration}$ from 0 to 2500 with steps of 50 and minimized the MAE between $GrxA_{model,calibrated}$ and $GrxA_{experimental}$. For a given value of $k_{GrxA,calibration}$, the MAE was estimated from the P*grxA*-SCFP3 intensity values of the cells at the open end of the growth trenches in M9 glucose media at steady-state treated with various concentrations of H_2O_2 (12.5 μM, 25 μM, 37.5 μM, 50 μM, 62.5 μM, 75 μM, 82.5 μM, 100 μM and 500 μM).

$$MAE = \left|\frac{GrxA_{experimental,i} - GrxA_{model,calibrated,i}}{GrxA_{experimental,i}}\right|$$

Where i denotes the different H_2O_2 concentrations.

The MAE plotted against $k_{GrxA,calibration}$ showed a minimum at 1150 [Figures S3A and S3B].

General stress response model parametrisation

For the plots in Figure 3, we used the following parameter values to solve the general stress response model: $h_E = 1 a.u., K_{act} = 0.1 a.u. min^{-1}, h_{act} = 1\ a.u., K_{cat}$ was varied between 0 to $20 min^{-1}, R_{influx} = 1\ min^{-1}$, $[Toxin]_{external}$ was varied between 0 to 200 a.u. The parameters used in the growth model and cell-cell interaction model were the same as for the H_2O_2 stress response model.

Simulation input

Simulation runs were initialised by specifying the user-defined parameters: number of time points T, time step duration Δt (1 min), $[H_2O_2]_{external}$, time of H_2O_2 treatment (time point 50, unless mentioned otherwise), number of growth trenches (n_g), length of growth trenches (L_{trench}), initial cell lengths at t_0. Unless otherwise specified, the initial cell lengths were drawn from a random distribution to match experimental conditions where cells are loaded into growth trenches from an unsynchronised culture. Cells are positioned in a straight row with their poles touching. The values used for simulations are given in the Methods S1.

Simulation procedure

Simulating the interlinked cell-cell interaction model (I), stress response model (S), and growth model (G) required discretisation in space and time. The simulation procedure is shown schematically in Figure S1.

First, the cell-cell interaction model predicts the external $[H_2O_2]_{external}$ concentration that each cell is exposed to according to its position in the growth trench. The reaction-diffusion equation (Equation 9) is solved for the uptake of $[H_2O_2]_{external}$ by the outermost cell first which is exposed to the fixed concentration of $[H_2O_2]_{external}$ in the growth media. This leads to a reduced external $[H_2O_2]_{external}$ concentration for the cell located immediately beneath the outermost cell. The procedure is repeated to predict the external $[H_2O_2]_{external}$ concentration from one barrier cell to the next until the mother cell is reached at the bottom of the population.

Secondly, the stress response model predicts for each cell the changes in the intracellular $[H_2O_2]_{cell}$ concentration, concentration of reduced regulator $OxyR^{red}$, and concentrations of the stress response enzymes (GrxA, KatG, AhpCF) for the next time point (Equations 10, 11, 12, 13, and 14). It uses the external $[H_2O_2]_{external}$ concentration from the cell-cell interaction model and the cell elongation rate g (Equation 2) from the growth model as inputs for each cell.

Thirdly, the growth model uses the intracellular $[H_2O_2]_{cell}$ concentration from the stress response model as input to compute the cell elongation rate g for each cell (Equation 2), and changes the number, size, and positions of all the cells for the next time point (Equation 1). Cell elongation and division pushes cells towards the open end of the trench. If the summed cell lengths exceed the trench length then the outermost cell is removed at the next time point.

The outputs of the interdependent models at one time point are used as input conditions for the next time point. The simulation runs for a set number of time points. Parallel growth trenches are treated as independent simulation runs.

Simulation visualization

We generated videos to visualise the simulation results using custom Python code and the following libraries: Image, ImageDraw from PIL, tifffile. We drew rows of rod-shaped cells according to the outputs of the growth model and the intensity of each cell was given by a linear grayscale conversion of the GrxA value from the stress response model. Simulations with multiple growth trenches were combined into the same image and converted into .tiff files. The tiff files were concatenated over time into videos using Fiji.[52] Further data visualisation and extraction (e.g. lineage tracing) was performed on the simulated videos using BACMMAN software[53] as for experimental data, described above.

Model of the oxidative stress response with noise

Stochastic differential equations

The stress response model was modified using the Langevin approach[63–65] to account for stochasticity in gene regulation. This approach involves adding a stochastic noise term to the set of differential equations (Equations 10, 11, 12, 13, and 14). Specifically, for the deterministic differential equation of the form:

$$\frac{dX}{dt} = f(X) \qquad \text{(Equation 18)}$$

where $X = [OxyR]_{Red}, [GrxA], [KatG], [AhpC]$, we add a noise term η:

$$\frac{dX}{dt} = f(X) + \eta \qquad \text{(Equation 19)}$$

$$\eta = \sigma \frac{\mathrm{dW}}{\mathrm{dt}}$$

Where W is the variable of a Weiner process satisfying the condition that $W_t - W_0 \sim \mathcal{N}$.[66] $\mathcal{N}$ is a normal distribution with mean of μ and variance of σ^2. Rearranging Equation 17 gives the stochastic differential equation (SDE) as follows:

$$dX = f(X)dt + \sigma dW \qquad \text{(Equation 20)}$$

Euler-Maruyama method

To solve Equation 20 numerically, we use the Euler-Maruyama method.[67,68] Let $\Delta t = t_{i+1} - t_i$ where i represents the iteration step from 0,1, … to N (number of iterations). Therefore, Equation 20 can be rewritten as:

$$X_{i+1} = X_i + f(X_i)\Delta t + \sigma Z_i \sqrt{\Delta t} \qquad \text{(Equation 21)}$$

Where Z_i represents the normal distribution (Weiner process derivatives) with mean = 0 and variance = 1.

Noisy equations for oxidative stress response

To solve the equations numerically, the equations were non-dimensionalised in time $t_{nd} = t \cdot g$, where g is the elongation rate. Then, noise term γ_{gene} was added to each equation, giving the following:

$$\frac{d[OxyR]_{Red}}{dt_{nd}} = \frac{1}{g}\left(- K_{ox}[OxyR_{Red}][H_2O_2]_{cell} + K_{red}[GrxA]\left(\frac{[OxyR]_{total} - [OxyR]_{Red}}{([OxyR]_{total} - [OxyR]_{Red}) + h_{OxyR}}\right)\right) + \gamma_{OxyR} \qquad \text{(Equation 22)}$$

$$\frac{d[GrxA]}{dt_{nd}} = \frac{1}{g}\left(R_{grxA,basal} + K_{grxA,act}\left(\frac{[OxyR]_{total} - [OxyR]_{Red}}{([OxyR]_{total} - [OxyR]_{Red}) + h_{grxA,act}}\right) - g[GrxA]\right) + \gamma_{GrxA} \qquad \text{(Equation 23)}$$

$$\frac{d[KatG]}{dt_{nd}} = \frac{1}{g}\left(R_{katG,basal} + K_{katG,act}\left(\frac{[OxyR]_{total} - [OxyR]_{Red}}{([OxyR]_{total} - [OxyR]_{Red}) + h_{katG,act}}\right) - g[KatG]\right) + \gamma_{KatG} \qquad \text{(Equation 24)}$$

$$\frac{d[AhpC]}{dt_{nd}} = \frac{1}{g}\left(R_{aphC,basal} + K_{ahpC,act}\left(\frac{[OxyR]_{total} - [OxyR]_{Red}}{([OxyR]_{total} - [OxyR]_{Red}) + h_{ahpC,act}}\right) - g[AhpC]\right) + \gamma_{AhpC} \quad \text{(Equation 25)}$$

To solve the equations numerically using the Euler-Maruyama method, Equations 22, 23, 24, and 25 were converted to the form shown in Equation 18, giving the following:

$$[OxyR]_{Red\,i+1} = [OxyR]_{Red\,i} + \frac{\Delta t_{nd}}{g}\left(- K_{ox}[OxyR]_{Red\,i}[H_2O_2]_{cell\,i} + K_{red}[GrxA]_i\left(\frac{[OxyR]_{total} - [OxyR]_{Red\,i}}{([OxyR]_{total} - [OxyR]_{Red\,i}) + h_{OxyR}}\right)\right) + \sigma_{oxyR}Z_i\sqrt{\Delta t_{nd}} \quad \text{(Equation 26)}$$

$$[GrxA]_{i+1} = [GrxA]_i + \frac{\Delta t_{nd}}{g}\left(R_{grxA,basal} + K_{grxA,act}\left(\frac{[OxyR]_{total} - [OxyR]_{Red\,i}}{([OxyR]_{total} - [OxyR]_{Red\,i}) + h_{grxA,act}}\right) - g[GrxA]_i\right) + \sigma_{GrxA}Z_i\sqrt{\Delta t_{nd}} \quad \text{(Equation 27)}$$

$$[KatG]_{i+1} = [KatG]_i + \frac{\Delta t_{nd}}{g}\left(R_{katG,basal} + K_{katG,act}\left(\frac{[OxyR]_{total} - [OxyR]_{Red\,i}}{([OxyR]_{total} - [OxyR]_{Red\,i}) + h_{katG,act}}\right) - g[KatG]_i\right) + \sigma_{KatG}Z_i\sqrt{\Delta t_{nd}} \quad \text{(Equation 28)}$$

$$[AhpC]_{i+1} = [AhpC]_i + \frac{\Delta t_{nd}}{g}\left(R_{aphC,basal} + K_{katG,act}\left(\frac{[OxyR]_{total} - [OxyR]_{Red\,i}}{([OxyR]_{total} - [OxyR]_{Red\,i}) + h_{ahpC,act}}\right) - g[AhpC]_i\right) + \sigma_{AhpC}Z_i\sqrt{\Delta t_{nd}} \quad \text{(Equation 29)}$$

The noise term is added in the form $\gamma_{gene} = \sigma_{gene}Z_i\sqrt{\Delta t_{nd}}$

For our simulations $\Delta t_{nd} = 5\cdot 10^{-6}\ min^{-1}$ and $\{\sigma_{OxyR}, \sigma_{GrxA}, \sigma_{KatG}, \sigma_{AhpC}\} = \{0.0002, 0.02, 0.02, 0.02\}$.

We chose values of σ such that the magnitude of the fluctuations of the stochastic response model (S*) was similar to the magnitude of the chaotic fluctuations of the full deterministic model (S+G+I). MAE was computed between the CV (Coefficient of variation) of GrxA from the deterministic model and the stochastic stress response model solved for a range of concentrations of H_2O_2 for σ of [0.00005, 0.0001,0.0005, 0.001,0.005, 0.01,0.05, 0.1].

$$MAE = \left|\frac{CV_{deterministic} - CV_{stochastic}}{CV_{deterministic}}\right|$$

This model was then coupled with the deterministic G and I models individually (S*+G, S*+I) or with both G and I models (S*+G+I), as required.

Current Biology, Volume *33*

Supplemental Information

Chaos in a bacterial stress response

Divya Choudhary, Kevin R. Foster, and Stephan Uphoff

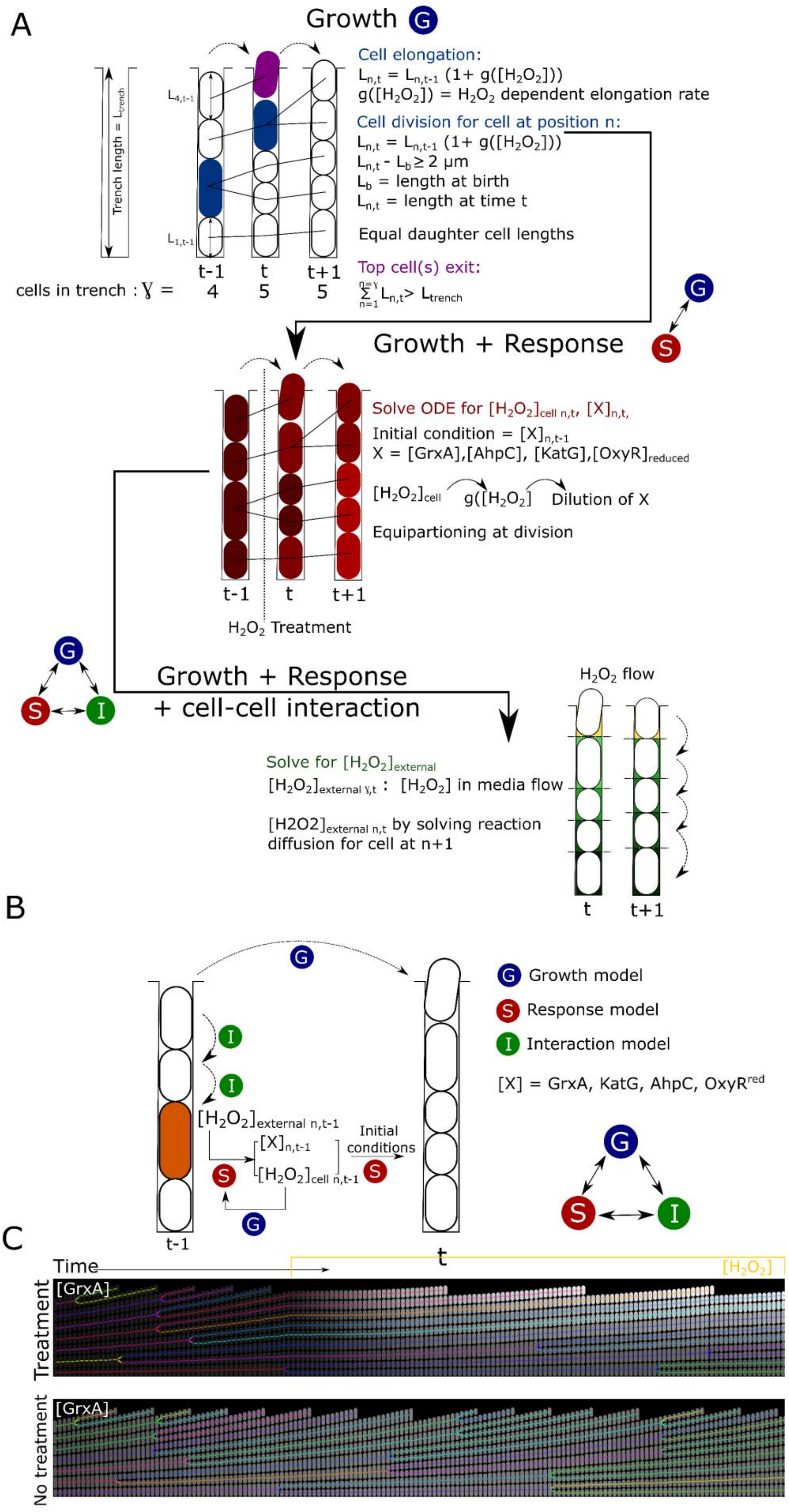
A
Growth G
Cell elongation:
L_{n,t} = L_{n,t-1} (1+ g([H2O2]))
g([H2O2]) = H2O2 dependent elongation rate
Cell division for cell at position n:
L_{n,t} = L_{n,t-1} (1+ g([H2O2]))
L_{n,t} - L_b ≥ 2 μm
L_b = length at birth
L_{n,t} = length at time t
Equal daughter cell lengths
Top cell(s) exit:
Trench length = L_trench
cells in trench : ɣ = 4 5 5
t-1 t t+1
Growth + Response
Solve ODE for [H2O2]_{cell n,t}, [X]_{n,t},
Initial condition = [X]_{n,t-1}
X = [GrxA],[AhpC], [KatG],[OxyR]_reduced
[H2O2]_cell g([H2O2] Dilution of X
Equipartioning at division
H2O2 Treatment
Growth + Response + cell-cell interaction
Solve for [H2O2]_external
[H2O2]_{external ɣ,t} : [H2O2] in media flow
[H2O2]_{external n,t} by solving reaction diffusion for cell at n+1
H2O2 flow
B
Growth model
Response model
Interaction model
[X] = GrxA, KatG, AhpC, OxyR^red
[H2O2]_{external n,t-1}
[X]_{n,t-1}
[H2O2]_{cell n,t-1}
Initial conditions
C
Time
[H2O2]
[GrxA]
Treatment
No treatment

Figure S1. Illustration of model simulation with and without H_2O_2 treatment. Related to Figure 1

Illustration of the iterative procedure for simulating the inter-dependent growth, stress response, and cell-cell interaction models. (**A**) The growth model predicts the changes in the number, size, and positions of the cells in the 1-dimensional cell population for the next time point in the simulation. The output of the growth model feeds into the stress response model, which predicts for each cell the changes in the intracellular concentration of H_2O_2, concentration of reduced regulator $OxyR^{red}$, and concentrations of the stress response enzymes (GrxA, KatG, AhpCF) for the next time point. The cell elongation rate from the growth model predicts the dilution rate of enzymes. At steady-state, the average enzyme production rates and H_2O_2 influx rate are balanced with the respective average dilution rates. Temporary unbalance between production and dilution rates leads to fluctuations in concentrations in single cells. The cell-cell interaction model predicts the external H_2O_2 concentration that each cell is exposed to. A reaction-diffusion equation is solved for the uptake of H_2O_2 by the outermost cell first which is exposed to the fixed concentration of H_2O_2 in the growth media. This leads to a reduced external H_2O_2 concentration for the cell located immediately beneath the outermost cell. The procedure is repeated to predict the external H_2O_2 concentration from one cell to the next until the mother cell is reached at the bottom of the population. The outputs of the interdependent models at one time point are used as input conditions for the next time point. The simulation runs for a set number of time points. (**B**) Schematic representation for solving the growth G, response S and cell-cell interaction I models simultaneously for a representative focal cell shown in orange. I computes the $[H_2O_2]_{external}$ for the focal cell. This feeds into S solving for the concentrations of different enzymes [X] that are regulated by OxyR (e.g. GrxA, KatG, AhpC, $OxyR_{red}$) and $[H_2O_2]_{cell}$. $[H_2O_2]_{cell}$ is used as input for G to modulate the elongation rate of cell. The outputs serve as initial condition for the next time point. (**C**) Representative kymographs produced by the model for a single 1-dimensional cell population tracked over time. Greyscale shows GrxA concentration with constant 260 µM H_2O_2 treatment (top; duration of treatment marked in yellow) and without treatment (bottom).

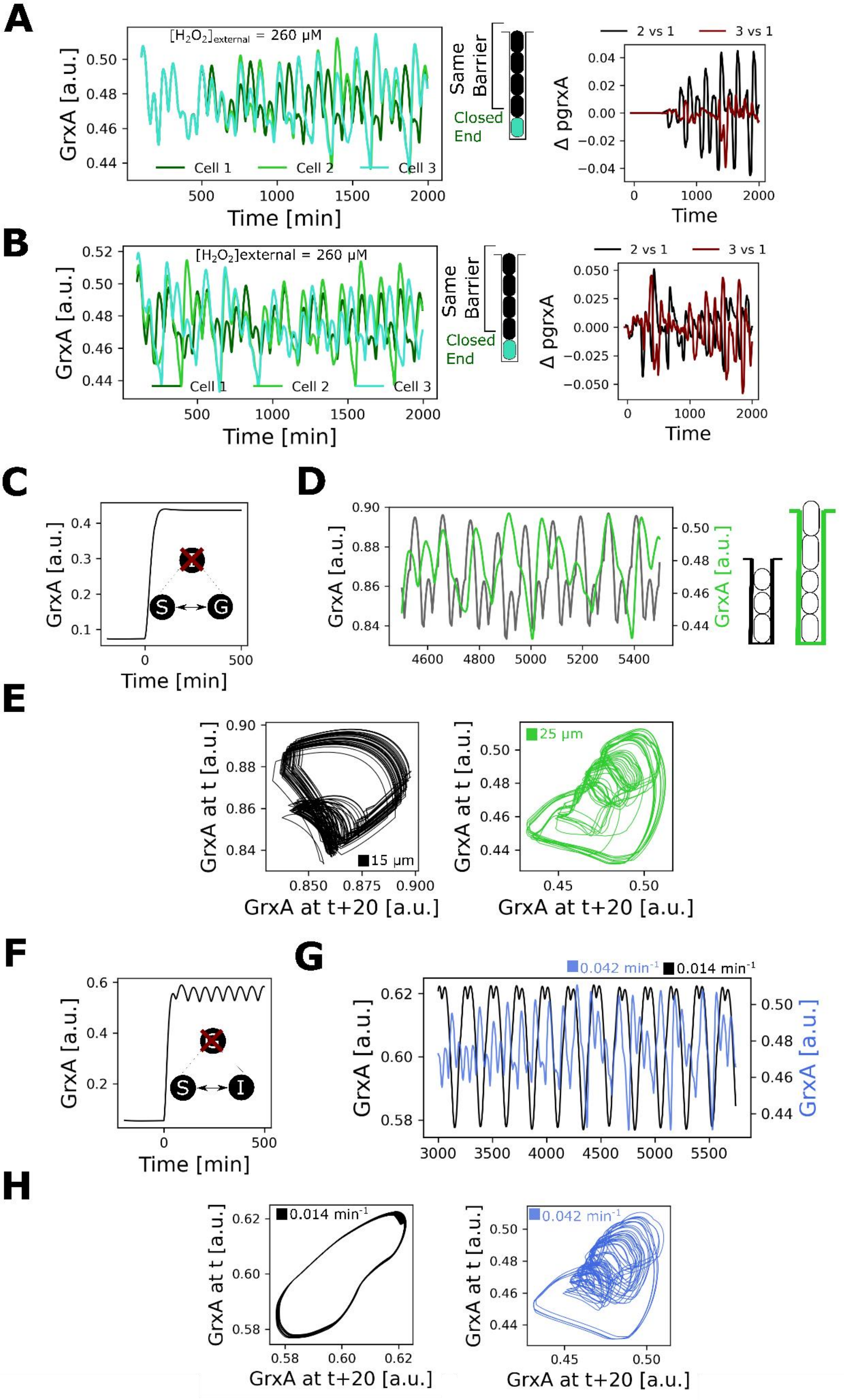

A
$[H_2O_2]_{external}$ = 260 µM
GrxA [a.u.]
Time [min]
Cell 1
Cell 2
Cell 3
Same Barrier
Closed End
2 vs 1
3 vs 1
Δ pgrxA
Time
B
$[H_2O_2]$external = 260 µM
C
GrxA [a.u.]
Time [min]
D
GrxA [a.u.]
E
GrxA at t [a.u.]
GrxA at t+20 [a.u.]
15 µm
25 µm
F
G
0.042 min⁻¹
0.014 min⁻¹
H

Figure S2. Chaotic traces of simulated stress response trajectories diverge with small differences in initial conditions and require strong coupling between stress response and cell-cell interactions or growth rate. Related to Figures 2 and 6.

(**A, B**) Left: Simulated trajectories of GrxA concentration in mother cells under constant 260 µM H_2O_2 treatment. The simulations were performed for mother cells that differ very slightly in their stage of the cell cycle at the start of the simulation. All other parameters and initial conditions were identical for the three simulations (including the number and sizes of the other cells in the population). (**A**) Mother cell length difference of $+2.5 \cdot 10^{-8}$ % (cell 2) and $+5 \cdot 10^{-8}$ % (cell 3). (**B**) Mother cell length difference of 2.5 % (cell 2) and 5% (cell 3). Right: The plots show the difference in GrxA concentration between mother cell pairs (cell 2 vs 1, cell 3 vs 1). The larger difference in initial conditions for panel B compared to panel A leads to a faster divergence of trajectories. (**C**) Response fluctuations are no longer chaotic if simulations are performed such that cell-cell interactions are absent. In this case, the concentration $[H_2O_2]_{external}$ is constant and identical for all cells in a trench. Plot shows steady GrxA expression for mother cell with 10 µM H_2O_2 treatment starting at t = 0 min. (**D**) Representative simulated GrxA traces of mother cells from the full S+G+I model (with cell-cell interactions causing $[H_2O_2]_{external}$ gradients). Periodic dynamics are seen for cells growing in a shorter growth trench with smaller population (15 µm, 2 – 4 cells, black) and chaotic dynamics for cells growing in a longer trench with larger population (25 µm, 5 – 7 cells, light green). (**E**) Phase diagrams of the GrxA traces in panel D showing closed orbits for periodic oscillations (left) but not for chaotic fluctuations (right). (**F**) Response fluctuations are no longer chaotic if simulations are performed such that growth rate is unaffected by H_2O_2 ($g([H_2O_2])$ is constant). This change uncouples the G model from the S and I models. Plot shows periodic oscillations in GrxA expression for mother cell with 400 µM H_2O_2 treatment starting at t = 0 min. (**G**) Representative simulated GrxA traces of mother cells from the full S-G-I model (with growth rate dependent on H_2O_2). Periodic dynamics are seen at low growth rate (0.014 min^{-1}, black) and chaotic dynamics at high growth rate (0.042 min^{-1}, light blue). (**H**) Phase diagrams of the GrxA traces in panel G showing closed orbits for periodic oscillations (left) but not for chaotic fluctuations (right).

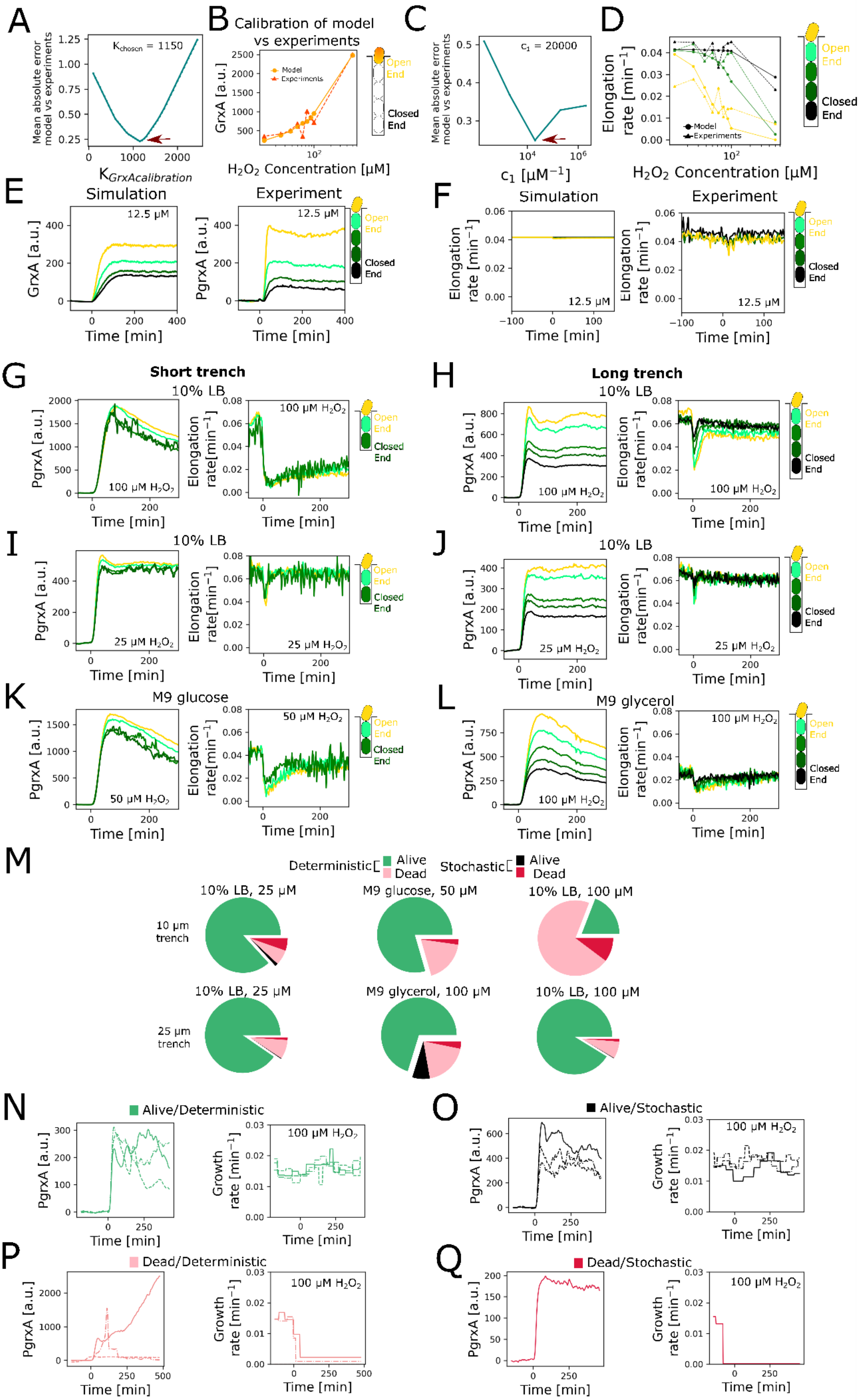
A
B Calibration of model vs experiments
C
D
E Simulation
Experiment
F Simulation
Experiment
G Short trench
10% LB
H Long trench
10% LB
I 10% LB
J 10% LB
K M9 glucose
L M9 glycerol
M
Deterministic Alive Dead
Stochastic Alive Dead
10% LB, 25 μM
M9 glucose, 50 μM
10% LB, 100 μM
10 μm trench
M9 glycerol, 100 μM
25 μm trench
N Alive/Deterministic
O Alive/Stochastic
P Dead/Deterministic
Q Dead/Stochastic
Time [min]
PgrxA [a.u.]
Elongation rate [min−1]
Growth rate [min−1]
H2O2 Concentration [μM]
Open End
Closed End

Figure S3. Stress response dynamics, elongation rate changes, and survival for different growth and H_2O_2 treatment conditions. Related to Figure 4 and 5.

Calibrating model parameters: (**A**) Mean absolute error for experimental P*grxA*-SCFP3 intensities versus GrxA concentration in model simulations for different values of calibration factor $\boldsymbol{k_{GrxA,calibration}}$. The arrow indicates the minimum point of the curve that was chosen as the parameter for all other simulations. (**B**) Experimental mean P*grxA*-SCFP3 (dashed dark orange; triangles) and calibrated GrxA values from the model (orange; circles) of cells at the open end of the trenches at steady-state for different H_2O_2 concentrations. (**C**) Mean absolute error of the cell elongation rate from experimental data versus model simulations for different values of calibration factor $\boldsymbol{c_1}$. The arrow indicates the minimum point of the curve that was used for parameter $\boldsymbol{c_1}$. (**D**) Mean cell elongation rates from model simulations (circles) and experiments (dashed; triangles) for cells at different positions in the trench over a range of H_2O_2 concentrations (yellow to black color represents cells from open to closed end of a growth trench). (**E, F**) Modelling predictions and experimental data for mean GrxA expression (left) and mean elongation rates (right) under constant 12.5 µM H_2O_2 treatment in M9 glucose from t=0 min for cells at different positions in growth trench (n = 20 simulated trenches and 3 experimental repeats). (**G-L**) Summary of oxidative stress response dynamics in experiments with different growth and H_2O_2 treatment conditions. Cells growing in shorter trenches have reduced population size, increased stress response expression, and prolonged inhibition of growth with H_2O_2 treatment compared to cells in long trenches. Mean P*grxA*-SCFP3 expression (left) and elongation rate (right) for cells growing in M9 glucose +10% LB in (**G**) 10 µm and (**H**) 25 µm trenches with 100 µM H_2O_2 treatment starting at t = 0 min (colour indicates cell position in the trench from yellow at open end to green/black at closed end of the trench, n = 3 experimental repeats). (**I, J**) Same as for panels G and H but with 25 µM H_2O_2 treatment starting at t = 0 min (n=3 experimental repeats). (**K, L**) Same as for panels G and H but with 50 µM H_2O_2 and 100 µM H_2O_2 treatment for cells growing in M9 glucose and M9 glycerol respectively (n = 3 experimental repeats). (**M**) Pie-charts indicate the fraction of dead (pink / red) and alive (green / black) mother cells classified as having deterministic (green / pink) or stochastic (black / red) stress response dynamics (based on P*grxA*-SCFP3 signal) for t > 60 min after start of H_2O_2 treatment according to the chaos decision tree algorithm by Toker *et al.* Cells were grown in different media (M9 glucose + 10% LB, M9 glucose, M9 glycerol) inside long (25 µm) or short (10 µm) growth trenches, treated with low (25 µM) or high (100 µM) H_2O_2 (on average 1249 ± 457 cells (std) with n ≥ 3 experimental repeats per pie-chart).

(**N-Q**) Representative traces of P*grxA*-SCFP3 (left) and growth rate (right) for mother cells in 25 μm growth trenches treated with 100 μM H_2O_2 at t = 0 min predicted as (**N**) alive and deterministic (green); (**O**) alive and stochastic (black); (**P**) dead and deterministic (pink); and (**Q**) dead and stochastic (red). For traces predicted as dead, the time of cell death is when growth rate drops abruptly to zero.

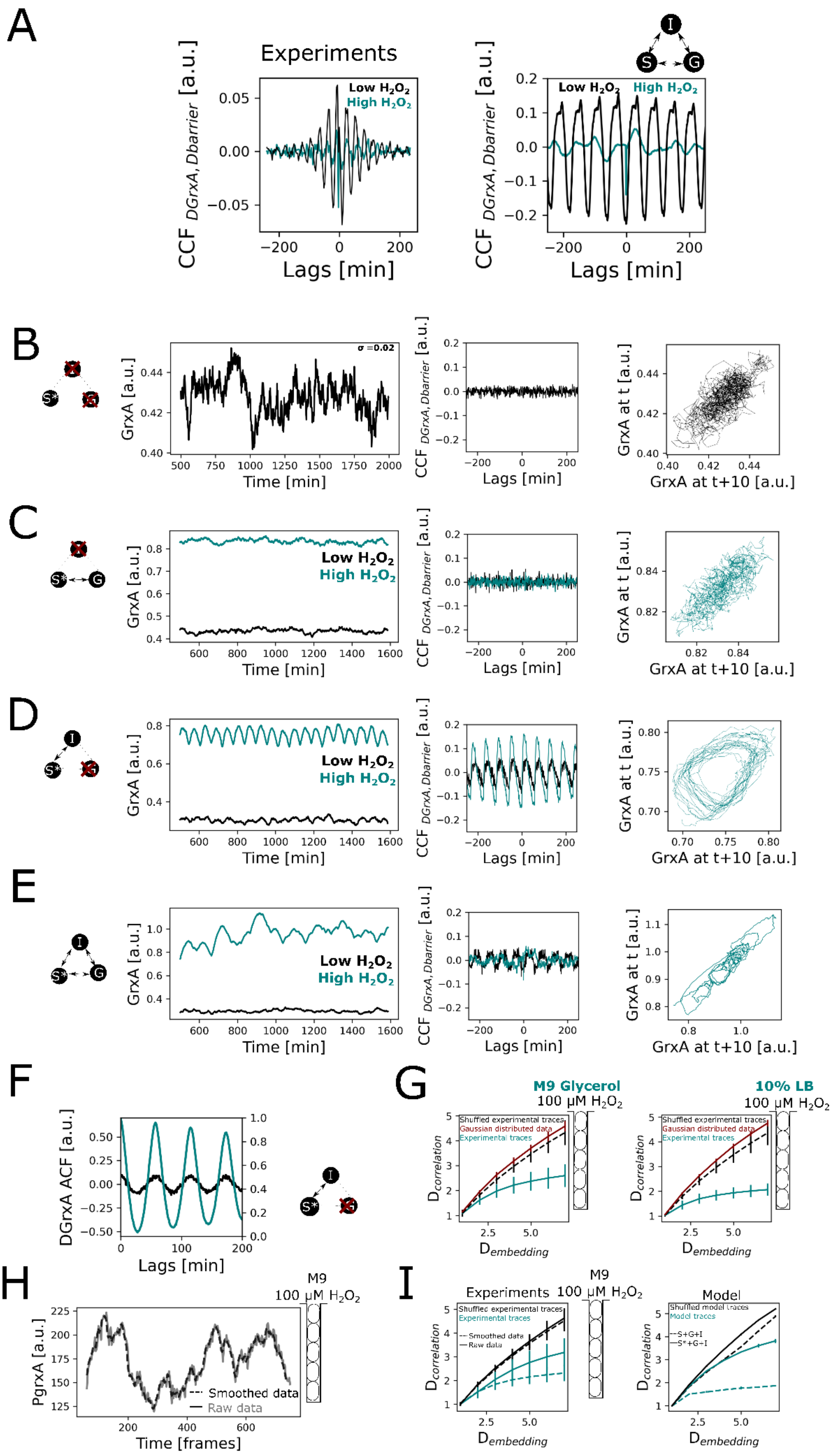
A
Experiments
CCF DGrxA, Dbarrier [a.u.]
Low H2O2
High H2O2
Lags [min]
I
S
G
B
σ =0.02
GrxA [a.u.]
Time [min]
GrxA at t [a.u.]
GrxA at t+10 [a.u.]
C
D
E
F
DGrxA ACF [a.u.]
Lags [min]
G
M9 Glycerol
100 μM H2O2
10% LB
Shuffled experimental traces
Gaussian distributed data
Experimental traces
Dcorrelation
Dembedding
H
M9
PgrxA [a.u.]
Smoothed data
Raw data
Time [frames]
I
Experiments
Model
Smoothed data
Raw data
Shuffled model traces
Model traces
S+G+I
S*+G+I

Figure S4. Response fluctuations are not driven by noise. Related to Figure 5.

(**A**) Response fluctuations are generated by a simple cyclic cell growth pattern that generates oscillations in the number of barrier cells. Fluctuations in GrxA traces for mother cells are anti-correlated with changes in the number of barrier cells over time. Mean cross-correlation between changes in GrxA (DgrxA) and number of barrier cells (Dbarrier) for mother cells in experiment (left) and model (right) for periodic (black, low H_2O_2) and chaotic (teal, high H_2O_2) conditions. Simulations of the stress response model with gene expression noise S* do not match experimental observations unless the response model S* is coupled with the growth G and cell-interactions I model components. Therefore, noise alone cannot explain the response fluctuations. (**B**) Noisy stress response model without growth and cell-interactions components does not show expected cross-correlations: Example simulated GrxA trace shows gene expression fluctuations (left), but no cross-correlation function (CCF) peaks are seen between changes in GrxA (DGrxA) and changes in barrier cells (Dbarrier) (middle), in disagreement with experiments. Corresponding phase diagram (right) does not show the characteristic extended orbits of periodic or chaotic fluctuations. **(C)** Coupling of the growth model with the noisy response model S*+G does not generate the expected cross-correlation and phase diagram orbits, neither for low (black) nor high (teal) H_2O_2 concentrations. **(D)** Coupling of the cell-interaction model with the noisy response model S*+I generates the expected cross-correlation and phase diagram orbits, but the autocorrelation of the dynamics for low (black) and high (teal) H_2O_2 concentrations is inconsistent with experiments. **(E)** Coupling of the growth and cell interaction models with the noisy response model S*+G+I generates the expected cross-correlation and phase diagram orbits for low (black) and high (teal) H_2O_2 concentrations. (**F**) For simulated traces of the S*+I model (panel D), the ACF shows periodic oscillations even at high H_2O_2 concentration (teal), in contrast to experiments where high H_2O_2 leads to chaos and loss of ACF peaks (Fig 6C). (**G**) Correlation dimension analysis using Grassberger – Procaccia algorithm indicates that observed fluctuations are deterministic. Curves show the correlation dimension as a function of the embedding dimension from P*grxA*-SCFP3 traces of mother cells during H_2O_2 treatment at steady-state under the indicated measurement conditions. Curves for experimental traces (teal) are compared to experimental traces with randomly shuffled time points (dashed black) and to synthetic traces with data points drawn from a Gaussian distribution with the same mean and standard deviation as the experimental traces (maroon). The curves from experimental traces saturate at a finite correlation dimension of ~2 for all measurement conditions, consistent with deterministic

dynamics, whereas the curves from the randomized controls do not saturate. (970, 733 traces for cells growing in M9 glycerol and M9 glucose + 10% LB respectively, curves represent mean values with error bars representing standard deviation). (**H**) Effect of moving-mean smoothing on correlation dimension analysis. Example raw P*grxA*-SCFP3 trace (grey) and after smoothing with a filter window of 9 minutes (black dashed). (**I**) (left) Correlation dimension versus embedding dimension from P*grxA*-SCFP3 traces as in panel H for raw data (solid lines) or with moving-mean smoothing (dashed lines). (right) Application of correlation analysis to simulated GrxA traces from the full deterministic model S+G+I (dashed lines) and the full model with noisy stress response S*+G+I (solid lines).

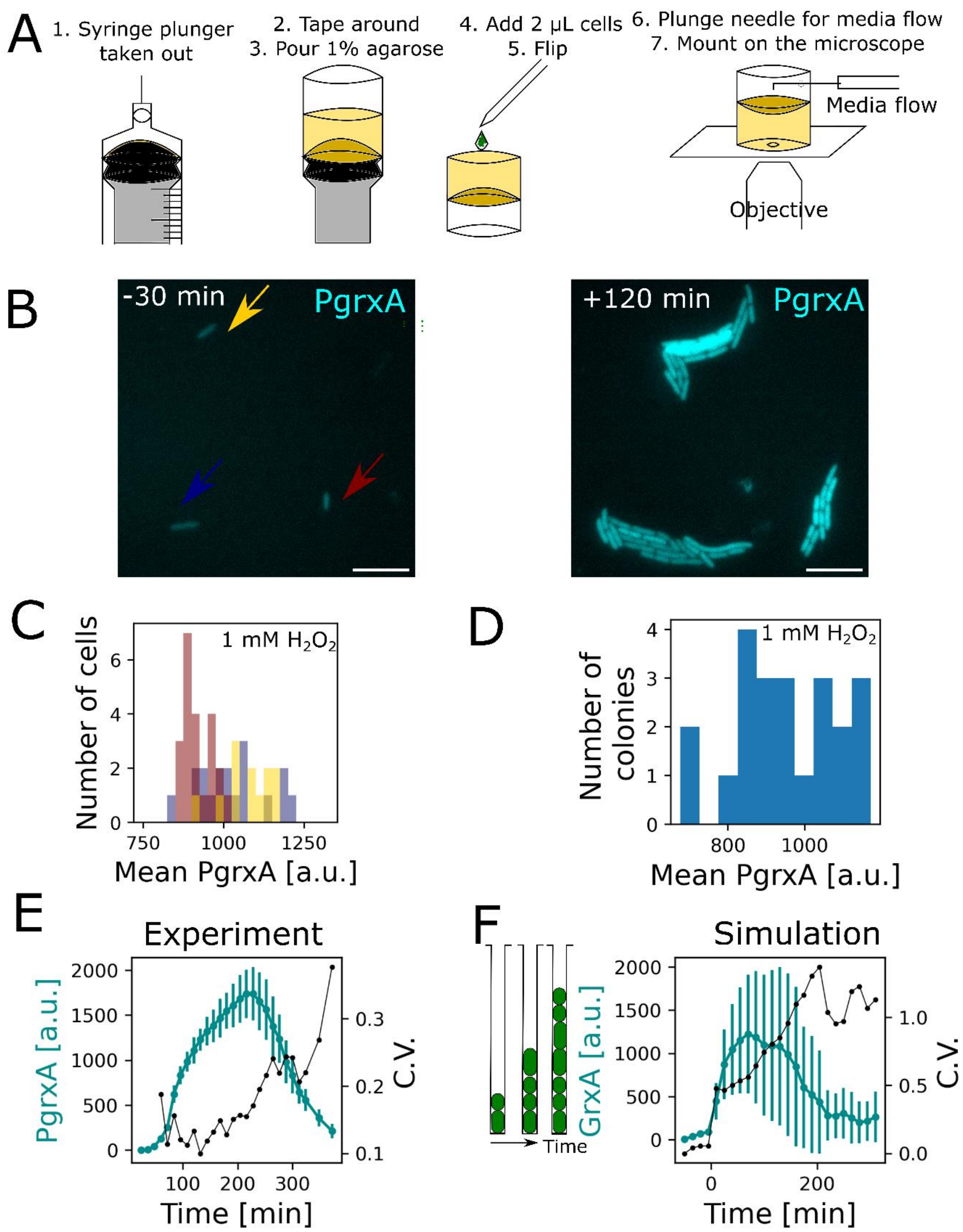

Figure S5. Bacterial colonies in experiments exhibit oxidative stress response fluctuations consistent with chaos. Related to Figure 5.

(**A**) Schematic describing the preparation of agarose pads for imaging bacterial microcolonies under continuous H_2O_2 infusion. (**B**) Snapshots of P*grxA*-SCFP3 expression of microcolonies growing from 3 single cells under 1 mM H_2O_2 treatment (left; 30 mins before treatment, right;

120 mins after treatment). (**C**) Histograms of P*grxA*-SCFP3 intensities of individual cells show heterogeneous responses at 120 minutes after start of 1 mM H_2O_2 treatment (colours corresponding to microcolonies arising from cells marked with arrows in panel B, n=54 cells). (**D**) Histograms of mean P*grxA*-SCFP3 intensities for entire colonies at 120 minutes after start of 1 mM H_2O_2 treatment showing variability in responses between different colonies (n = 22 colonies with 16 ± 12 cells per colony). (**E, F**) Oxidative stress response dynamics and cell-to-cell heterogeneity of microcolonies in experiments match model predictions. The GrxA expression (cyan lines, mean ± standard deviation across cells) initially increases upon constant 1 mM H_2O_2 treatment from t = 0 min but the average response of the whole population decreases again when a growing number of cells in the interior of the population becomes protected by the H_2O_2 scavenging activity of the cells at the edge of the colony. This divergence of the population response causes an increase over time in the coefficient of variation (C.V., black lines) of GrxA expression across cells in the colony. (**E**) Experimental results for P*grxA*-SCFP3 expression of all cells tracked in a microcolony over time (increasing from n = 2 to 737 cells over time). (**F**) Simulation of a one-dimensional population growing in a very long trench (100 µm) matches experimental results shown in panel E for microcolonies (n = 3 simulations).

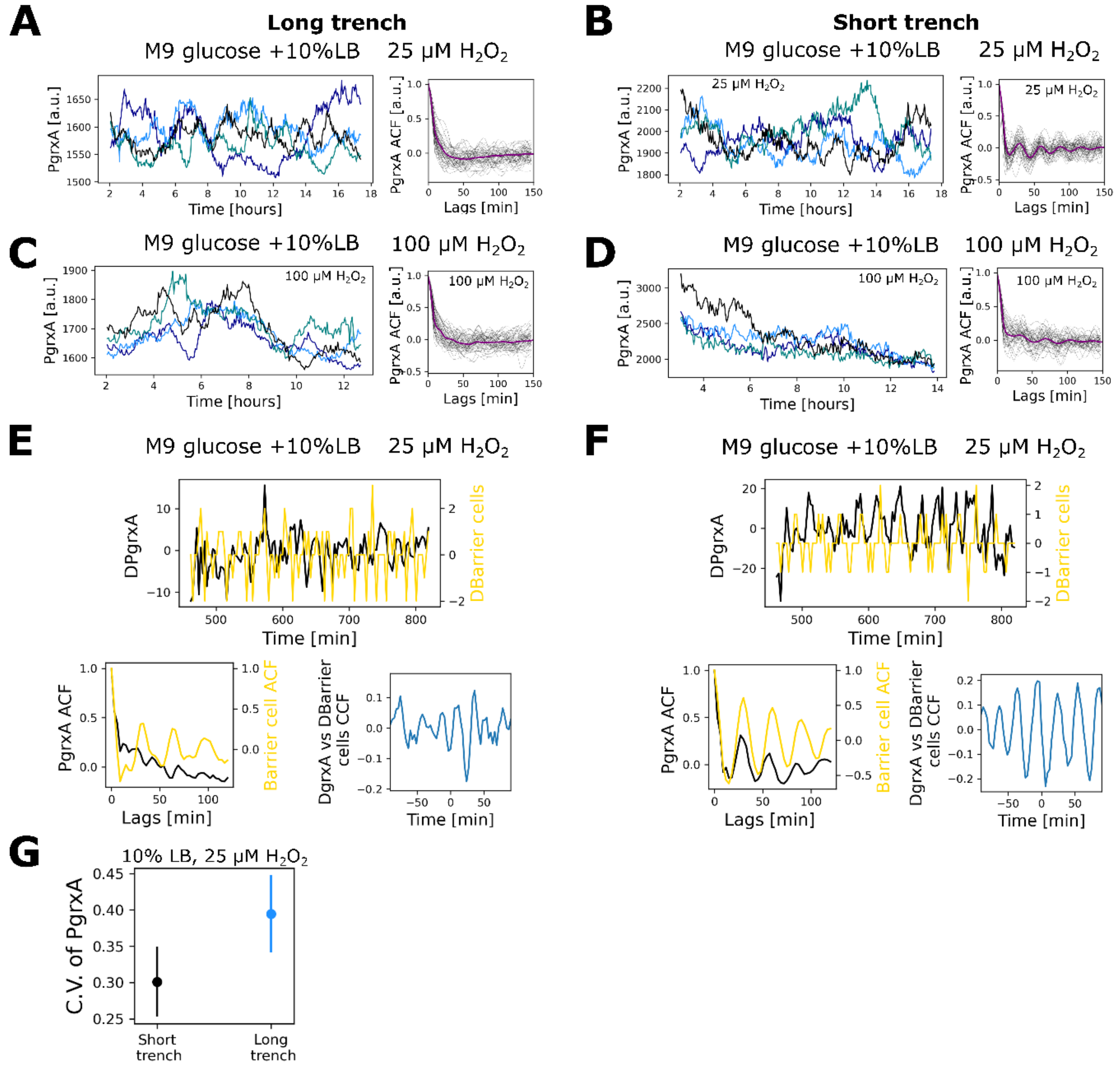

Figure S6. Deterministic chaos in oxidative stress response fluctuations at single cell level. Related to Figure 5 and 6.

Correlation analysis shows chaotic and periodic response dynamics at a single-cell level in experiments. (**A-D**) 5 representative P*grxA*-SCFP3 expression traces and ACF curves for 50 individual mother cells (black) and cell-average curve (purple). (**A**) Chaotic dynamics: M9 glucose + 10% LB with 25 µM H_2O_2 in 25 µm trenches. (**B**) Periodic dynamics: M9 glucose + 10% LB with 25 µM H_2O_2 in 10 µm growth trenches. (**C**) Chaotic dynamics: M9 glucose + 10% LB with 100 µM H_2O_2 in 25 µm trenches. (**D**) Periodic dynamics: M9 glucose + 10% LB with 100 µM H_2O_2 in 10 µm growth trenches. (**E, F**) Representative mother cell traces showing cross-correlation between changes in P*grxA*-SCFP3 expression (DpgrxA, black) and changes

in barrier cell numbers (DBarrier cells, yellow). Corresponding autocorrelation of DpgrxA (ACF, left) and cross-correlation of DpgrxA vs Dbarrier cells (CCF, right) plots for the single mother cell traces above. Cells growing in M9 glucose + 10% LB and treated with 25 µM H_2O_2 in (**E**) 25 µm trenches (chaotic dynamics) and (**F**) 10 µm trenches (periodic dynamics). (**G**) Under chaotic conditions cell-cell variability increases. Coefficient of variation (C.V.) of P*grxA*-SCFP3 expression for mother cells growing in M9 glucose + 10% LB and treated with 25 µM H_2O_2 in 25 µm (blue) and 10 µm trenches (black) (n= 3 experimental repeats, mean ± standard deviation).
